# Birth registration coverage according to the sex of the head of household: an analysis of national surveys from 93 low- and middle-income countries

**DOI:** 10.1186/s12889-022-14325-z

**Published:** 2022-10-19

**Authors:** Andrea Wendt, Franciele Hellwig, Ghada E Saad, Cheikh Faye, Ties Boerma, Aluisio J D Barros, Cesar G Victora

**Affiliations:** 1 International Center for Equity in Health, Postgraduate Program of Epidemiology, Federal University of Pelotas, Pelotas, Brazil; 2 International Center for Equity in Health, Post-Graduation Program in Epidemiology, Federal University of Pelotas, 1160 Marechal Deodoro St, 3rd floor., Pelotas, RS Brazil; 3 Faculty of Health Sciences, Department of Epidemiology and Population Health, American University of Beirut, Beirut, Lebanon; 4 African Population and Health Research Center, Nairobi, Kenya; 5 University of Manitoba, Winnipeg, Canada; 6 International Center for Equity in Health, Federal University of Pelotas, Pelotas, Brazil; 7 Programa de Pós-Graduação em Tecnologia em Saúde, Pontifícia Universidade Católica do Paraná, Curitiba, Brazil

**Keywords:** Health status disparities, Family characteristics, Birth certificates, Gender equity, Health equity

## Abstract

**Background:**

Within-country inequalities in birth registration coverage (BRC) have been documented according to wealth, place of residence and other household characteristics. We investigated whether sex of the head of household was associated with BRC.

**Methods:**

Using data from nationally-representative surveys (Demographic and Health Survey or Multiple Indicator Cluster Survey) from 93 low and middle-income countries (LMICs) carried out in 2010 or later, we developed a typology including three main types of households: male-headed (MHH) and female-led with or without an adult male resident. Using Poisson regression, we compared BRC for children aged less than 12 months living the three types of households within each country, and then pooled results for all countries. Analyses were also adjusted for household wealth quintiles, maternal education and urban-rural residence.

**Results:**

BRC ranged from 2.2% Ethiopia to 100% in Thailand (median 79%) while the proportion of MHH ranged from 52.1% in Ukraine to 98.3% in Afghanistan (median 72.9%). In most countries the proportion of poor families was highest in FHH (no male) and lowest in FHH (any male), with MHH occupying an intermediate position. Of the 93 countries, in the adjusted analyses, FHH (no male) had significantly higher BRC than MHH in 13 countries, while in eight countries the opposite trend was observed. The pooled analyses showed t BRC ratios of 1.01 (95% CI: 1.00; 1.01) for FHH (any male) relative to MHH, and also 1.01 (95% CI: 1.00; 1.01) for FHH (no male) relative to MHH. These analyses also showed a high degree of heterogeneity among countries.

**Conclusion:**

Sex of the head of household was not consistently associated with BRC in the pooled analyses but noteworthy differences in different directions were found in specific countries. Formal and informal benefits to FHH (no male), as well as women’s ability to allocate household resources to their children in FHH, may explain why this vulnerable group has managed to offset a potential disadvantage to their children.

**Supplementary Information:**

The online version contains supplementary material available at 10.1186/s12889-022-14325-z.

## Introduction

The 16th goal of the 2030 Agenda for Sustainable Development is focused on ensuring legal identity - including birth registration – for all individuals [1]. Birth registration is a human right and a guarantee that all children have a name, nationality and citizenship documents, thus allowing them to attend school and gain access to health services [2, 3]. Birth registration allows accurate measurement of age, which is essential for school admission, voting rights, military service and for being allowed to marry, drive, or consume alcohol. At national level, exact age measurements are important for policymaking, programming, planning and monitoring [3, 4].

While in many countries all children are registered at birth, in many poor countries this is not the rule [5]. The proportion of under-five children with birth registration ranges from 100% in most high income-countries to under 30%, particularly in some low-income African countries [6]. Although there is a global increase in birth registration coverage (BRC) [7], inequalities between and within countries remain, with children from rural areas and poor families being less likely to be registered [7]. While wealth-related and urban/rural disparities are often reported in the literature [7, 8], other dimensions of inequality are little explored as is the case for sex of the head of household, that could reveal gender inequality. An 2021 UNICEF report on obstacles women face in order to register the births of their children lists legislation that require the presence or consent by the father (with a few specific exemptions), barriers to register children born out of wedlock (proof of parents’ legal marriage), and cultural discriminatory practices identifying fathers as the primary responsible for the child [2]. Although such pre-requisites and absence of father are the most common barriers [9, 10], registration is also affected by distance from a facility (especially in rural areas), fees and other costs, bureaucracy, inefficiency and by lack of information about how obtain a certificate [10, 11].

Female-headed households are complex and context-dependent [12]. In most societies, they tend to be poorer than households headed by men [13, 14], which in addition to the above-described barriers could make registration difficult or even impossible in some settings. On the other hand, the literature suggests that children living in households headed by empowered may present favourable outcomes due to improved management of resources prioritizing the children, independently of poverty status, as well as being more likely to receive social assistance benefits that require proof of a child’s age [15].

Our search of the literature found four studies reporting on the association between female headship and birth registration. All studies were limited to a single country (two from Nigeria and one each from Uganda and India) and included sex of the head of household as one of several potential determinants of birth registration [4, 16–18]. Results from the literature are inconsistent, and there are no multicountry studies on this important issue.

Our goal is to address this data gap by describing BRC according to sex of the head of household in 93 low- and middle-income countries (LMICs). Our findings may help detect inequalities and identify vulnerable groups to be targeted in efforts to increase registration coverage.

## Methods

Our study relied upon the International Center for Equity in Health database (www.equidade.org), which includes the original data from publicly available child health surveys carried out since the mid-1990s, totalling more than 400 surveys for 121 countries. Nearly all surveys are Demographic and Health Survey (DHS) or Multiple Indicator Cluster Survey (MICS) with nationally representative samples and questionnaires focused on women in reproductive age. DHS and MICS are very comparable in terms of sampling, questionnaires, and protocols [19]; detailed information about it is found elsewhere [20, 21]. A relevant difference between these surveys refers to who is included as household members - while DHS includes visitors who slept in the house in the preceding night, MICS only includes usual residents. To attenuate this difference, we excluded visiting children from the DHS sample. Our analyses included the most recent dataset from all 93 countries with a survey carried out in 2010 or later with information on birth registration.

The initial set of analyses were carried out at individual level within each country dataset, examining the associations between birth registration for each child and the sex of the household head. In a second step, these results were pooled across countries.

### Household headship

Our main explanatory variable was the sex of head of household. DHS and MICS include a list of household members starting by the head of family, followed by information on the sex and age of each member. In DHS, information about sex of head of household and information on birth registration as well as covariates information are in the household members dataset. In MICS, the household information was merged with child dataset where the birth registration information is stored. Households without children were excluded from the analyses.

Based on this information, we classified sampled households according to sex of their heads. Because the simple classification into male or female -headed household (MHH or FHH) is oversimplified, we explored more granular definitions of subtypes of FHH. Our exploratory analyses divided FHHs into 16 subgroups according with the presence in the household of the woman’s husband, of another man aged 18 years or older, and of other women and children [12]. The frequencies of some subgroups were small in many countries, and our final typology was restricted to three categories: (a) male-headed household (MHH); (b) FHH with any adult male; (c) FHH without a male. This typology is described in detail in a previous publication [12, 22].

### Birth registration coverage

The outcome under study was the BRC, expressed as a proportion. Although the standard denominator for BRC includes all children aged less than five years, we opted to restrict the analyses to children under one year of age to present a more recent estimate, given that there was no information on how long the current head of household had been in this position. The numerator was children under one year who had been registered with civil authorities, with or without birth certificates.

### Covariates

We included three covariates in the individual-level adjusted analyses: wealth quintiles, maternal education (none/primary/secondary or more) and area of residence (urban/rural). These covariates were chosen based on the literature on child health and female-headed households in LMICs [2, 15, 22, 23].

Regarding wealth quintiles, the questionnaires collect information on household appliances (such as televisions, refrigerators, and other appliances), characteristics of the building (materials used for the walls, floor and roof, and presence of electricity, water supply and sanitary facilities), and other variables related to economic status (ownership of the house, vehicles, land or livestock). In each dataset, these variables were included in principal component analysis (PCA) for all households in the sample, excluding variables that are only relevant for one domain (e.g. livestock or land size which only apply to rural areas). Next, two separate PCAs were carried out for urban and rural households, including all relevant variables in each domain. Using linear regression procedures, the urban and rural PCA results are combined into a single asset index, which may then be split into quintiles [24–26].

### Individual-level statistical analyses

Our analyses comprise two sets of results. The first is an individual level analysis within each country, with children (and their households) as the units of analysis. The descriptive analyses were aimed at describing the distribution of households in each country according to sex of the head, describing socioeconomic positions of each category of households, showing BRC at national level and for each category of households. These analyses were followed by calculation of BRC ratios comparing the three categories, still within each country. The second set of findings we present include pooled analyses of these country-specific results, aimed at summarizing the BRC ratios observed in the 93 countries.

For the individual level analysis, children were assigned to a category of household headship, either male-headed (MHH), female-headed with an adult male present (FHH any male) or female-headed without an adult male (FHH no male). To assess differences in socioeconomic position among these groups, we estimated the proportion of poor families (defined as those in the first and second quintiles of wealth) in each of them (Supplementary Figure 1). Next, we estimated BRC for each of the three household headship categories within each country. Equiplots (https://www.equidade.org/equiplot) were used for graphical representation of inequalities. The dots in equiplots represent BRC in each group of households while the lines represent the differences in percentage points among the highest and lowest coverage groups.

We then calculated crude and adjusted BRC prevalence ratios for the two FHH groups compared to MHH using Poisson regression with robust variance. For each country, two prevalence ratio estimates were obtained, one for FHH (any male) versus MHH, and another for FHH (no male) versus MHH. Poisson regression has the advantage of producing prevalence ratio estimates, which are more easily interpretable than the odds ratios derived through logistic regression. This is especially relevant when the outcome is common like BRC. For example, if BRC is 90% in one group and 60% in the reference category, the prevalence ratio will be 1.5 while the odds ratio will be 6.0. Although Poisson regression was originally developed for count outcomes, since 2003 it has been increasingly used for outcomes expressed as proportions because adjusting the standard errors with robust estimation allows prevalence ratios and their confidence intervals to be assessed [27, 28]. All our estimates are reported with respective 95% confidence intervals and Wald tests were used to compare BRC between each FHH category and MHH. Adjusted analyses aimed at assessing whether other household characteristics could explain observed crude effects of headship.

### Pooled analyses

To obtain pooled results across study countries, we used a two-step random effects approach. First, estimates of prevalence ratios and their standard errors were obtained for each country as described above. Second, pooled estimates across all countries were obtained by weighing country-specific prevalence ratios inversely by their standard errors, using a two-step meta-analytic approach [29] through the *metan* command in Stata. The analytical approach is commonly used in meta-analyses of separate studies, with the only difference being that the prevalence ratios had been generated in our own individual-level analyses. The random effects approach accounts for heterogeneity among countries. The I^2^ statistic was used to measure heterogeneity, reflecting the percentage of total variation that is due between country variation in effect [30]. I^2^ values below 25% are usually considered low, between 25% and 75% moderate and values above 75% are considered high [30]. To assess whether prevalence ratios varied according to BRC levels, we repeated the pooled analyses after stratifying countries into terciles of BRC based on the ranking of all countries included in our study.

All analyses were carried out with Stata (StataCorp. 2019. Stata Statistical Software: Release 16. College Station, TX: StataCorp LLC.) considering the sample design (clustering, weights and strata). We also presented the coverage ratios for each country in Supplementary Table 5. Anonymized data from MICS and DHS surveys are publicly available and the institutions responsible for carrying out these surveys were responsible for ethical clearance.

## Results

Surveys carried out in 2010 or later were available for 93 countries, including 28 low-income-, 40 lower-middle- and 25 upper-middle-income countries. These represent 90.3%, 75.5% and 44.6%, respectively, of all world countries in each income group. The total number of children studied was 210,796 (median = 1,535; Interquartile range 739–2553) (Supplementary Table 1).

### Individual-level analyses

**Fig. 1:**
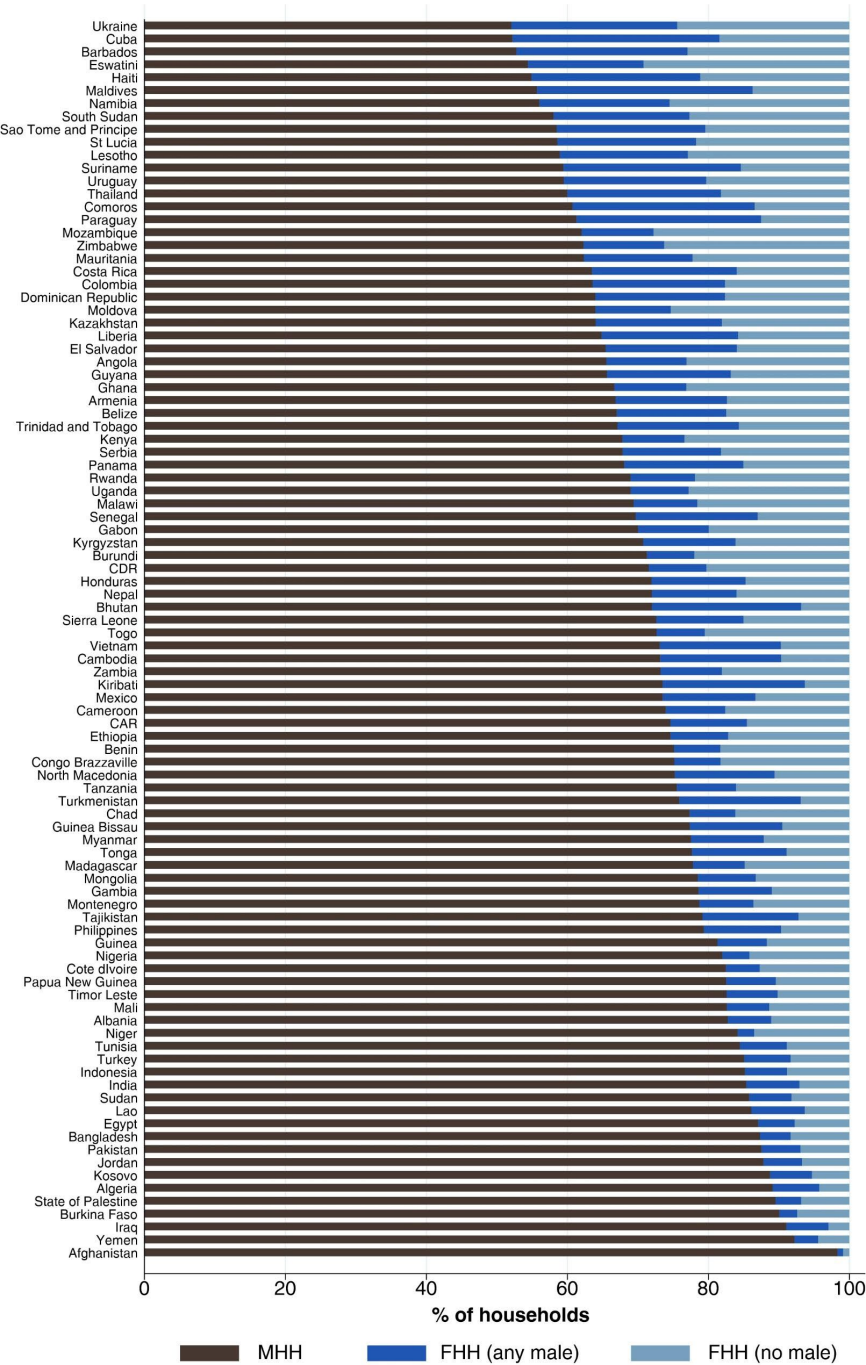
**Household headship distribution by country** Ordered by the proportion of male-headed households Number of countries: 93; Number of households: 211,306

In all figures, countries are ranked according to national BRC, ranging from 2.2% in Ethiopia to 100% in Thailand. Figure 1 and Supplementary Table 1 show household headship distribution by country. MHH ranged from 98.3% in Afghanistan to 52.1% in Ukraine, with a median of 72.9%. Four countries had over 25% of households in the FHH (any male) category: the Maldives (30.6%), Cuba (29.3%), Paraguay (26.2%), and Comoros (25.8%). Five countries had over 25% of all households in the FHH (no male) above 25%: Eswatini (29.2%), Mozambique (27.8%), Zimbabwe (26.3%), Namibia (25.5%), and Moldova (25.3%). Supplementary Fig. 1 presents the proportion of poor households (in the first and second quintiles of wealth) according to sex of head. There is considerable variability in the socioeconomic position of households headed by men and women, but for most countries the proportion of poor families is highest in FHH (no male) and lowest in FHH (any male) households, with the MHH group occupying an intermediate position.

**Fig. 2:**
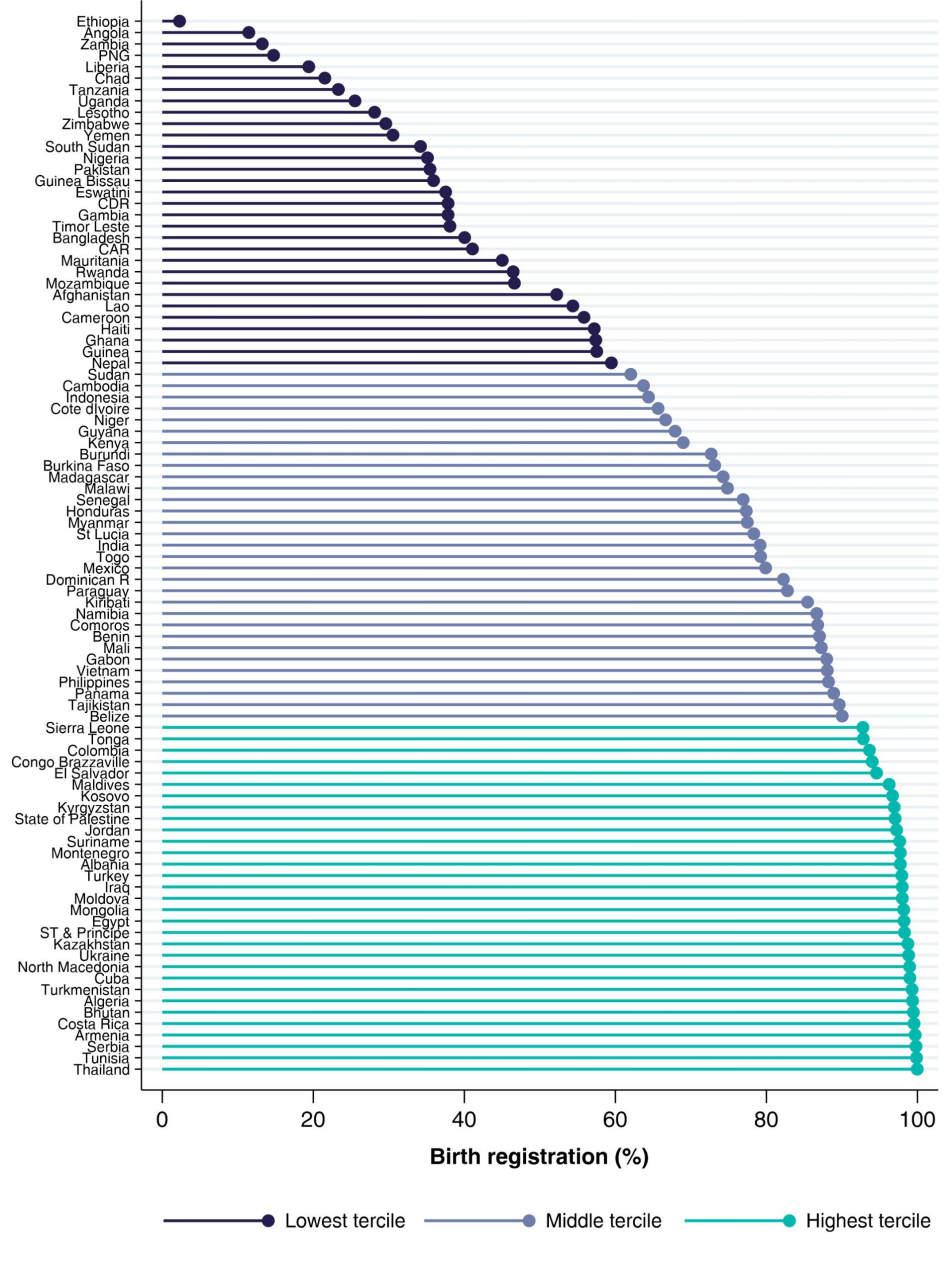
**Birth registration coverage by country** Ordered by birth registration coverage N of countries: 93; N of children:210,796

Figure 2 shows national BRC levels. Seven countries had coverage below 25%: Ethiopia (2.2%), Angola (11.5%), Zambia (13.2%), Papua New Guinea (14.7%), Liberia (19.4%), Chad (21.5%) and Tanzania (23.3%).

**Fig. 3:**
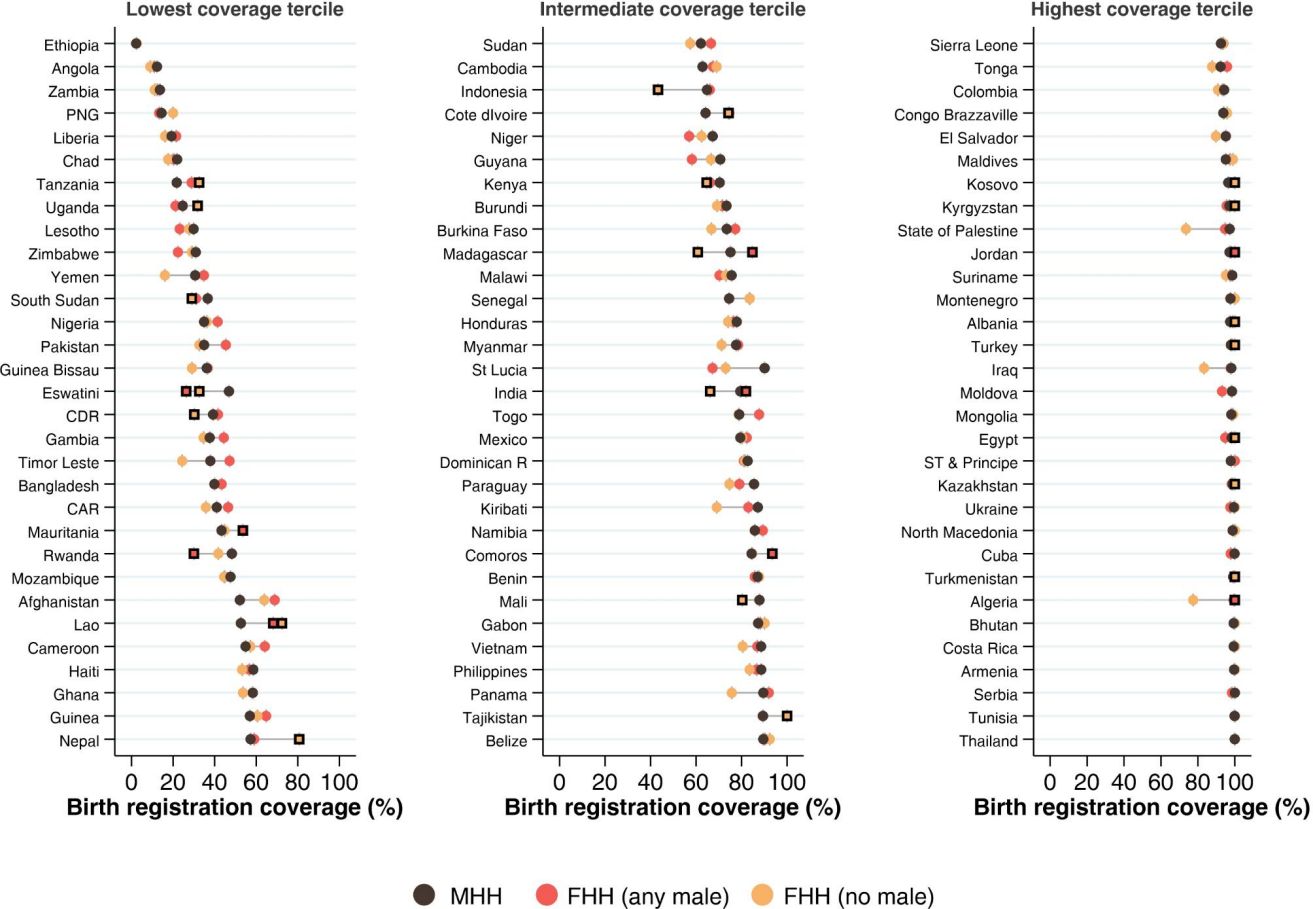
**Adjusted birth registration coverage according to household types** Square symbols identify FHH groups that are significantly (P < 0.05) different from the MHH group. Circles identify FHH groups for which the differences from MHH were not significant Countries with fewer than 25 children in the FHH (any male) group: Kosovo, Montenegro, St Lucia, State of Palestine and Tunisia. Countries with fewer than 25 children in the FHH (no male) group: Afghanistan, Algeria, Armenia, Iraq, Jordan, Kiribati, Kosovo, Kyrgyzstan, Montenegro, North Macedonia, Serbia, St Lucia, State of Palestine, Tonga, Turkey, Turkmenistan, and Vietnam Number of countries: 93; Number of children: 187,234

Supplementary Fig. 2 and Supplementary Table 2 show unadjusted BRC levels according to the three types of households derived from individual-level analyses. When BRC in FHH groups was significantly different from MHH, the circles are replaced by squares in Fig. 3. Regarding differences in BRC between MHH and FHH (no male), 13 of the 93 countries had higher BRC in FHH (no male) than in MHH whereas eight countries had differences in the opposite direction. For FHH (any male), nine countries had higher BRC than MHH and two countries had lower coverage.

The next step in the individual-level analyses included adjustment for wealth, maternal education and area of residence (Fig. 3 and Supplementary Table 3). Twelve countries (Albania, Congo Brazzaville, Egypt, Kazakhstan, Kosovo, Kyrgyzstan, Lao, Nepal, Papua New Guinea, Tajikistan, Tanzania and Turkey) showed higher coverage in FHH (no male) than in MHH, while the reverse (higher BRC in MHH) was observed in three countries (Burkina Faso, India and Madagascar). Regarding FHH (any male), five countries (Algeria, Comoros, Jordan, Kosovo and Turkmenistan) showed higher coverage in FHH (any male) than in MHH, and two countries (Eswatini and Guyana) showed the opposite trend (higher BRC in MHH).

Supplementary Table 4 lists the countries where the observed differences changed after adjustment and respective directions of associations. Supplementary Table 5 shows the crude and adjusted BRC coverage ratios by country.

**Table 1:** Pooled birth registration coverage ratios for FHH households compared to MHH in 93 countries. Results stratified by national terciles of birth registration coverage

	FHH (any male) compared to MHH				FHH (no male) compared to MHH				N of countries
**Terciles of birth registration coverage**	**Crude**	**I** ^**2**^	**Adjusted**	**I** ^**2**^	**Crude**	**I** ^**2**^	**Adjusted**	**I** ^**2**^	**Number of countries**
**Lowest tercile**	1.02 (0.95; 1.10)	55.6%	0.96 (0.90; 1.01)	28.2%	0.96 (0.89; 1.04)	64.8%	0.98 (0.92; 1.05)	49.8%	31
**Middle tercile**	1.01 (0.99; 1.03)	30.9%	0.99(0.97; 1.01)	25.3%	0.96 (0.91; 1.00)	85.1%	0.97 (0.93; 1.00)	69.3%	31
**Upper tercile**	1.01 (1.00; 1.02)	50.9%	1.00 (1.00; 1.01)	28.6%	1.01 (1.01; 1.01)	68.2%	1.01 (1.01; 1.01)	63.1%	31
**All countries**	1.01 (1.00; 1.01)	48.2%	1.00 (1.00; 1.01)	27.5%	1.01 (1.00; 1.01)	76.5%	1.01 (1.00; 1.01)	62.0%	93

Adjustment: wealth quintiles, area of residence and maternal education Reference: MHH

### Pooled analyses

With countries as the units of analysis, the pooled results are presented in Table 1. The pooled BRC ratio for FHH (any male) relative to MHH was equal to 1.01 (95% CI: 1.00; 1.01), indicating very similar coverage levels in these two groups when results were pooled across the 93 countries. For FHH (no male) relative to MHH, the pooled coverage ratio was also 1.01 (95% CI: 1.00; 1.01), again, showing no evidence of a consistent difference in BRC between FHH and MHH when all countries were grouped. The I^2^ statistics indicate moderate to high degrees of between-country heterogeneity in coverage ratios. Table 1 also shows pooled coverage ratios for each tercile of BRC, confirming the absence of consistent differences in countries with different coverage levels.

## Discussion

The proportion of FHH by countries varied widely, ranging from 1.7 to 47.8%. BRC among infants also varied markedly, from 2.2% in Ethiopia to 100% in Thailand. In general, BRC was not associated with sex of the head of household except for few countries, in most of which FHH without an adult male presented higher coverages than MHH.

There are few published studies evaluating the associations between sex of the head of household and BRC, that may be compared with our results. The presence and direction of the associations vary from country to country, a finding that mirrors our own results. A study from India, using the same DHS dataset as in our analyses, showed that BRC in FHH was 77.2%, while in MHH was 80.6% (p = 0.001) [16]. In contrast, three studies from Uganda and Nigeria did not find any difference between coverage in MHH and FHH [4, 17, 18]. It is important to highlight that all the above studies relied upon dichotomous classifications of household headship (MHH vs. FHH). In India, for example, we found that FHH (no male) had lower coverage than MHH, and in contrast FHH (any male) had higher coverage than the other two categories. Our finding highlights the variability between the two FHH groups in this country, and the importance of treating these separately.

Although studies assessing the relationship between FHH and birth registration are scarce, other studies exploring associations with child health and nutrition outcomes also showed high variability in the results [4, 22, 31]. There are at least two main explanations for the lack of consistent results. First, empowered woman who are heading a household may have greater bargaining power in the family, thus prioritizing the allocation of resources to their children [15]. On other hand, households without a male head tend to be poorer due to lower income or lack of land rights, and consequently be more vulnerable than MHH [32–34]. These aspects emphasise the complexity of the pathways between different types of FHH and child outcomes, suggesting that not only poverty status, but also gender, social and cultural norms may affect the associations in different directions. The importance of context cannot be overstated.

Our analyses of household socioeconomic position showed that FHH were an adult man was present were often wealthier than MHH, while FHH without an adult male are usually poorer than MHH. Adult males in FHH could be relatives of the head, such as children or younger brothers, or could be partners who are not regarded as the head for a number of reasons. In any case, adult males may contribute to the family income or ensure land rights, thus explaining the higher socioeconomic position of such households. In selected countries, FHH without an adult male may benefit from informal (friends, church, community and relatives) or formal assistance (governmental and NGO resources) [35–37]. Specifically regarding birth registration, a review of literature showed that governmental financial incentives play an important role in increasing of coverages, with increases of up 20%. For example, cash transfer programs may require birth certificates for enrolling children, thus promoting birth registration [38]. Although a country-by-country analyses are beyond the scope of the present study, we detected higher coverage of birth registration among FHH (no male) than in MHH in 13 countries, whereas differences in the opposite direction were observed in eight countries. This might be a consequence of such formal and informal incentives,[38] which may be investigated in further studies. In spite of differences being present in selected countries, our overall findings suggest that there is no consistent association when all countries are considered.

We found that adjustment for covariates that are strongly associated with BRC (maternal education, wealth index and area of residence) [7, 8, 16] did not result in marked changes from what had been observed in the crude analyses. Of the 13 countries with higher coverage among FHH (no male) than MHH, ten remained significant in the adjusted analyses. This suggests that possible effects of having a woman as the head of the household go beyond the effects of poverty, education or residence.

The lack of association between sex of the head of household and BRC in most countries could result from coverage being driven by structural features in these settings. Studies from Uganda and Lao, for example, found that children delivered in government hospitals had higher probability of being registered [18, 39] than those born elsewhere. Costs to birth registration are also cited as major reasons for non-registration. In Indonesia, a survey on barriers to birth registration identified that 51% of sample reported high costs as the main problem, followed by distance to place of registration (19%) and by lack of information on the necessary arrangements (15%) [10]. In Guinea-Bissau the main barriers were lack of required documentation (42%) and absence of the father (28%) – although it is worth noting that in our analyses there was no association in this country [9]. In Tanzania, a study with mixed methods identified that 96.3% of the women who delivered in two hospitals received a notification form when the child was born, but 45% of them wrongly assumed that this form was the actual certificate [11]. The in-depth interviews in this study showed that women consider the registration process complex and costly [11].

Our study has some limitations. First, one or both FHH groups are infrequent in some countries resulting in small samples and low statistical power. For some countries where we found significant associations, BRC was close to 100% in all groups, and the practical relevance of the differences is questionable. The lack of detailed information about household headship also is a limitation. For example, one cannot assess how long the woman has been in the position of head, and if her status is recent, it might not have yet reflected in birth registration; we tried to minimize this possibility by restricting the analyses to children under one year of age. Furthermore, the question about who the head is extremely subjective and may be interpreted in differently depending on community (e.g., it could be defined as the oldest person in some cultures and as the bread winners in others) [32, 40]. The pooled results should be interpreted with due caution because of presence of important heterogeneity, which reflecting important variability from country to country. Lastly, adjusted analysis was restricted to children with information on all covariates (wealth index, area of residence and maternal education).

The strengths of this study are the inclusion of 93 low- and middle- income countries in the analysis with national representative surveys, which as far as we know is the largest set of such analyses. Previous studies were all were restricted to single countries. In addition, the use of a more granular typology of FHH, rather than a simple dichotomy, allowed a more detailed characterization of such a complex group. This is supported by the fact that, in comparison with MHH, FHH (no male) households tended to be poorer than MHH, whereas FHH (any male) tended to be wealthier, supporting the notion that FHH are not necessarily a more vulnerable group than MHH.

## Conclusion

In summary, sex of the head of household was not associated with BRC in most countries studied. FHH without an adult male tended to be the poorest group in most countries, yet showed higher BRC than MHH in 13 countries, while the reverse was observed in only eight countries. These findings suggest that women who are heads of household often manage to offset their family’s socioeconomic vulnerability and to be as likely – if not more likely – to register their children as those from households headed by men. Further research is needed to identify country-specific structural variables affecting BRC (such as hospital practices, requirements from cash transfer programs, direct and indirect costs of registration, complexity of registration requirements and other barriers). Universal child registration is a human right [2, 5] and monitoring inequalities in coverage are a useful tool to promote change.

## Electronic supplementary material

Below is the link to the electronic supplementary material.

Supplementary Material 1

## Data Availability

All data generated or analysed during this study are included in this published article and its supplementary information files.

## Declarations

All methods were performed in accordance with the relevant guidelines and regulations. DHS and MICS surveys request country-level ethical review and approval. All data used were anonymized and informed consent statements were obtained from all participants in each country. Data analysis for this study is from publicly available datasets and further ethical approval is not required.
