# Supplementary material for: Birth registration coverage according to the sex of the head of household: an analysis of national surveys from 93 low- and middle-income countries
Source: BMC Public Health. 2022 Oct 19;22:1942. doi: 10.1186/s12889-022-14325-z (PMC9583473; doi:10.1186/s12889-022-14325-z)
Supplement: Supplementary file 1 — Supplementary Material 1 [file 12889_2022_14325_MOESM1_ESM.docx]

# Supplementary material

**Supplementary Table 1. Description of birth registration coverage and groups of household by country.**

| **Country** | **Year** | **Source** | **MHH** | **FHH (any male)** | **FHH (no male)** | **Birth registration** | | | |
| --- | --- | --- | --- | --- | --- | --- | --- | --- | --- |
|  |  |  | **%** | **%** | **%** | **%** | **ll** | **ul** | **N of children** |
| Afghanistan | 2015 | DHS | 98.3 | 0.8 | 0.9 | 52.2 | 48.9 | 55.5 | 156.6 |
| Albania | 2017 | DHS | 82.7 | 6.2 | 11.1 | 97.7 | 95.4 | 98.9 | 292 |
| Algeria | 2018 | MICS | 89.1 | 6.6 | 4.3 | 99.4 | 98.8 | 99.6 | 297.8 |
| Angola | 2015 | DHS | 65.5 | 11.4 | 23.1 | 11.5 | 9.9 | 13.2 | 34.6 |
| Armenia | 2015 | DHS | 66.8 | 15.8 | 17.3 | 99.7 | 97.9 | 100 | 297.6 |
| Bangladesh | 2019 | MICS | 87.3 | 4.3 | 8.3 | 40 | 38.4 | 41.7 | 120.1 |
| Belize | 2015 | MICS | 67 | 15.5 | 17.5 | 90 | 86.1 | 92.9 | 269 |
| Benin | 2017 | DHS | 75.1 | 6.6 | 18.3 | 87 | 85.2 | 88.7 | 260.9 |
| Bhutan | 2010 | MICS | 72 | 21.1 | 6.8 | 99.5 | 98.5 | 99.8 | 297.8 |
| Burkina_Faso | 2010 | DHS | 90.1 | 2.5 | 7.4 | 73.1 | 70.8 | 75.4 | 219.3 |
| Burundi | 2016 | DHS | 71.3 | 6.7 | 22 | 72.7 | 70.1 | 75.1 | 217.9 |
| CAR | 2018 | MICS | 74.6 | 10.9 | 14.5 | 41.1 | 38 | 44.3 | 123.4 |
| Cambodia | 2014 | DHS | 73.2 | 17.2 | 9.7 | 63.7 | 60 | 67.3 | 191 |
| Cameroon | 2018 | DHS | 74 | 8.4 | 17.6 | 55.8 | 52.5 | 59.2 | 167.5 |
| Chad | 2019 | MICS | 77.3 | 6.5 | 16.2 | 21.5 | 19.5 | 23.6 | 64.6 |
| Colombia | 2015 | DHS | 63.6 | 18.8 | 17.7 | 93.6 | 92.4 | 94.7 | 280.7 |
| Comoros | 2012 | DHS | 60.7 | 25.8 | 13.4 | 86.8 | 83.1 | 89.8 | 259.7 |
| Congo_Brazzaville | 2014 | MICS | 75.2 | 6.5 | 18.3 | 94 | 92.6 | 95.1 | 281.7 |
| Congo_Democratic_Republic | 2017 | MICS | 71.5 | 8.2 | 20.3 | 37.8 | 33.8 | 42 | 113.6 |
| Costa_Rica | 2011 | MICS | 63.5 | 20.5 | 16 | 99.6 | 96.9 | 99.9 | 296.4 |
| Cote_dIvoire | 2016 | MICS | 82.5 | 4.8 | 12.7 | 65.7 | 62.1 | 69.1 | 196.9 |
| Cuba | 2019 | MICS | 52.2 | 29.3 | 18.4 | 99 | 94.1 | 99.8 | 292.9 |
| Dominican_Republic | 2014 | MICS | 64 | 18.4 | 17.6 | 82.3 | 80.1 | 84.2 | 246.6 |
| Egypt | 2014 | DHS | 87.1 | 5.2 | 7.8 | 98.2 | 97.4 | 98.8 | 294.4 |
| El_Salvador | 2014 | MICS | 65.4 | 18.6 | 16 | 94.6 | 92.3 | 96.2 | 283.1 |
| Eswatini | 2014 | MICS | 54.4 | 16.4 | 29.2 | 37.5 | 32.6 | 42.8 | 112.9 |
| Ethiopia | 2016 | DHS | 74.6 | 8.2 | 17.2 | 2.3 | 1.6 | 3.3 | 7.2 |
| Gabon | 2012 | DHS | 70 | 10 | 20 | 88 | 84.5 | 90.8 | 263.3 |
| Gambia | 2018 | MICS | 78.6 | 10.4 | 11 | 37.8 | 34.8 | 41 | 113.6 |
| Ghana | 2017 | MICS | 66.7 | 10.2 | 23.1 | 57.4 | 54.3 | 60.4 | 172.1 |
| Guinea | 2018 | DHS | 81.3 | 7 | 11.7 | 57.5 | 54.1 | 60.9 | 172.5 |
| Guinea_Bissau | 2018 | MICS | 77.4 | 13.1 | 9.5 | 35.9 | 33 | 38.9 | 107.8 |
| Guyana | 2014 | MICS | 65.6 | 17.6 | 16.8 | 67.9 | 62.9 | 72.6 | 203.4 |
| Haiti | 2016 | DHS | 54.9 | 23.9 | 21.1 | 57.2 | 54 | 60.4 | 171.6 |
| Honduras | 2011 | DHS | 71.9 | 13.3 | 14.7 | 77.3 | 75 | 79.5 | 231.8 |
| India | 2015 | DHS | 85.4 | 7.5 | 7.1 | 79.2 | 78.7 | 79.7 | 237.6 |
| Indonesia | 2017 | DHS | 85.2 | 6 | 8.9 | 64.4 | 62.2 | 66.5 | 193.1 |
| Iraq | 2018 | MICS | 91.1 | 6 | 3 | 98 | 97.2 | 98.5 | 293.7 |
| Jordan | 2017 | DHS | 87.8 | 5.5 | 6.7 | 97.2 | 95.9 | 98.2 | 291.3 |
| Kazakhstan | 2015 | MICS | 64 | 17.9 | 18.1 | 98.7 | 97.8 | 99.3 | 295.8 |
| Kenya | 2014 | DHS | 67.8 | 8.8 | 23.4 | 69 | 66.9 | 71 | 206.9 |
| Kiribati | 2018 | MICS | 73.5 | 20.2 | 6.3 | 85.4 | 81.3 | 88.8 | 255.5 |
| Kosovo | 2019 | MICS | 88.8 | 5.9 | 5.3 | 96.7 | 93.9 | 98.3 | 288.9 |
| Kyrgyzstan | 2018 | MICS | 70.8 | 13.1 | 16.2 | 96.9 | 94.6 | 98.3 | 289.8 |
| Lao | 2017 | MICS | 86.1 | 7.6 | 6.3 | 54.4 | 51.4 | 57.3 | 163.1 |
| Lesotho | 2018 | MICS | 58.9 | 18.1 | 22.9 | 28.1 | 23.1 | 33.7 | 84.9 |
| Liberia | 2013 | DHS | 64.9 | 19.4 | 15.8 | 19.4 | 15.8 | 23.6 | 58.8 |
| Madagascar | 2018 | MICS | 77.8 | 7.3 | 14.9 | 74.3 | 72 | 76.4 | 222.7 |
| Malawi | 2015 | DHS | 69.4 | 9.1 | 21.6 | 74.8 | 72.8 | 76.8 | 224.4 |
| Maldives | 2016 | DHS | 55.7 | 30.6 | 13.7 | 96.3 | 92.8 | 98.1 | 287.2 |
| Mali | 2018 | DHS | 82.6 | 6 | 11.4 | 87.3 | 85.1 | 89.2 | 261.6 |
| Mauritania | 2015 | MICS | 62.3 | 15.4 | 22.3 | 45 | 41.7 | 48.4 | 135.1 |
| Mexico | 2015 | MICS | 73.5 | 13.2 | 13.3 | 79.9 | 75 | 84 | 238.9 |
| Moldova | 2012 | MICS | 64 | 10.7 | 25.3 | 98 | 95.8 | 99 | 292.8 |
| Mongolia | 2018 | MICS | 78.5 | 8.2 | 13.3 | 98.2 | 96.6 | 99 | 293.8 |
| Montenegro | 2013 | MICS | 78.7 | 7.7 | 13.6 | 97.7 | 94.2 | 99.1 | 291 |
| Mozambique | 2015 | DHS | 62 | 10.2 | 27.8 | 46.6 | 41.8 | 51.5 | 139.9 |
| Myanmar | 2015 | DHS | 77.5 | 10.3 | 12.1 | 77.5 | 73.7 | 80.9 | 232.1 |
| Namibia | 2013 | DHS | 56 | 18.5 | 25.5 | 86.7 | 83.7 | 89.1 | 259.5 |
| Nepal | 2019 | MICS | 72 | 12 | 16 | 59.5 | 55.5 | 63.3 | 178.3 |
| Niger | 2012 | DHS | 84.1 | 2.4 | 13.5 | 66.6 | 63.3 | 69.8 | 199.7 |
| Nigeria | 2018 | DHS | 82 | 3.8 | 14.2 | 35.1 | 33.2 | 37.1 | 105.4 |
| North_Macedonia | 2018 | MICS | 75.2 | 14.1 | 10.6 | 99 | 97 | 99.7 | 295.7 |
| Pakistan | 2017 | DHS | 87.5 | 5.5 | 6.9 | 35.4 | 31.2 | 39.9 | 106.5 |
| Panama | 2013 | MICS | 68.1 | 16.9 | 15 | 88.9 | 85.4 | 91.7 | 266 |
| Papua_New_Guinea | 2016 | DHS | 82.5 | 7 | 10.4 | 14.7 | 12.1 | 17.8 | 44.6 |
| Paraguay | 2016 | MICS | 61.3 | 26.2 | 12.5 | 82.8 | 78.7 | 86.2 | 247.7 |
| Philippines | 2017 | DHS | 79.4 | 11 | 9.7 | 88.2 | 85.5 | 90.5 | 264.2 |
| Rwanda | 2014 | DHS | 69 | 9.1 | 21.9 | 46.5 | 43.5 | 49.5 | 139.5 |
| Sao_Tome_and_Principe | 2019 | MICS | 58.5 | 21.1 | 20.5 | 98.3 | 95.3 | 99.4 | 293 |
| Senegal | 2019 | DHS | 69.7 | 17.3 | 13 | 76.9 | 73.1 | 80.4 | 230.4 |
| Serbia | 2019 | MICS | 67.8 | 14 | 18.2 | 99.8 | 98.6 | 100 | 298.4 |
| Sierra_Leone | 2019 | DHS | 72.6 | 12.4 | 15 | 92.8 | 91.3 | 94 | 278.1 |
| South_Sudan | 2010 | MICS | 58 | 19.3 | 22.7 | 34.2 | 31.3 | 37.2 | 102.7 |
| St_Lucia | 2012 | MICS | 58.6 | 19.7 | 21.8 | 78.3 | 64 | 88 | 230.3 |
| State_of_Palestine | 2019 | MICS | 89.5 | 3.6 | 6.9 | 97 | 95.8 | 97.9 | 290.7 |
| Sudan | 2014 | MICS | 85.8 | 6 | 8.2 | 62 | 58.7 | 65.3 | 186 |
| Suriname | 2018 | MICS | 59.4 | 25.2 | 15.4 | 97.7 | 95.6 | 98.8 | 292.1 |
| Tajikistan | 2017 | DHS | 79.1 | 13.6 | 7.2 | 89.6 | 87.3 | 91.6 | 268.5 |
| Tanzania | 2015 | DHS | 75.5 | 8.4 | 16.1 | 23.3 | 20.8 | 26.1 | 70.2 |
| Thailand | 2019 | MICS | 60 | 21.8 | 18.2 | 100 | 99.8 | 100 | 299.8 |
| Timor_Leste | 2016 | DHS | 82.6 | 7.3 | 10.2 | 38.1 | 34.8 | 41.5 | 114.4 |
| Togo | 2017 | MICS | 72.7 | 6.8 | 20.5 | 79.2 | 75.4 | 82.6 | 237.2 |
| Tonga | 2019 | MICS | 77.7 | 13.4 | 8.9 | 92.8 | 87.3 | 96 | 276.1 |
| Tunisia | 2018 | MICS | 84.5 | 6.6 | 8.9 | 99.9 | 99.2 | 100 | 299.1 |
| Turkey | 2013 | DHS | 85.1 | 6.6 | 8.3 | 97.9 | 96.1 | 98.9 | 292.9 |
| Turkmenistan | 2019 | MICS | 75.9 | 17.2 | 6.9 | 99.3 | 98.1 | 99.7 | 297.1 |
| Uganda | 2016 | DHS | 69 | 8.2 | 22.8 | 25.5 | 23.5 | 27.6 | 76.6 |
| Ukraine | 2012 | MICS | 52.1 | 23.5 | 24.4 | 98.8 | 95.4 | 99.7 | 293.9 |
| Vietnam | 2013 | MICS | 73.1 | 17.2 | 9.7 | 88.1 | 85 | 90.5 | 263.6 |
| Yemen | 2013 | DHS | 92.2 | 3.4 | 4.4 | 30.5 | 28 | 33.2 | 91.7 |
| Zambia | 2018 | DHS | 73.2 | 8.7 | 18.1 | 13.2 | 11.3 | 15.4 | 39.9 |
| Zimbabwe | 2019 | MICS | 62.3 | 11.5 | 26.3 | 29.6 | 26.5 | 32.8 | 88.9 |

**Supplementary Figure 1. Socioeconomic description of households according to sex of head.**


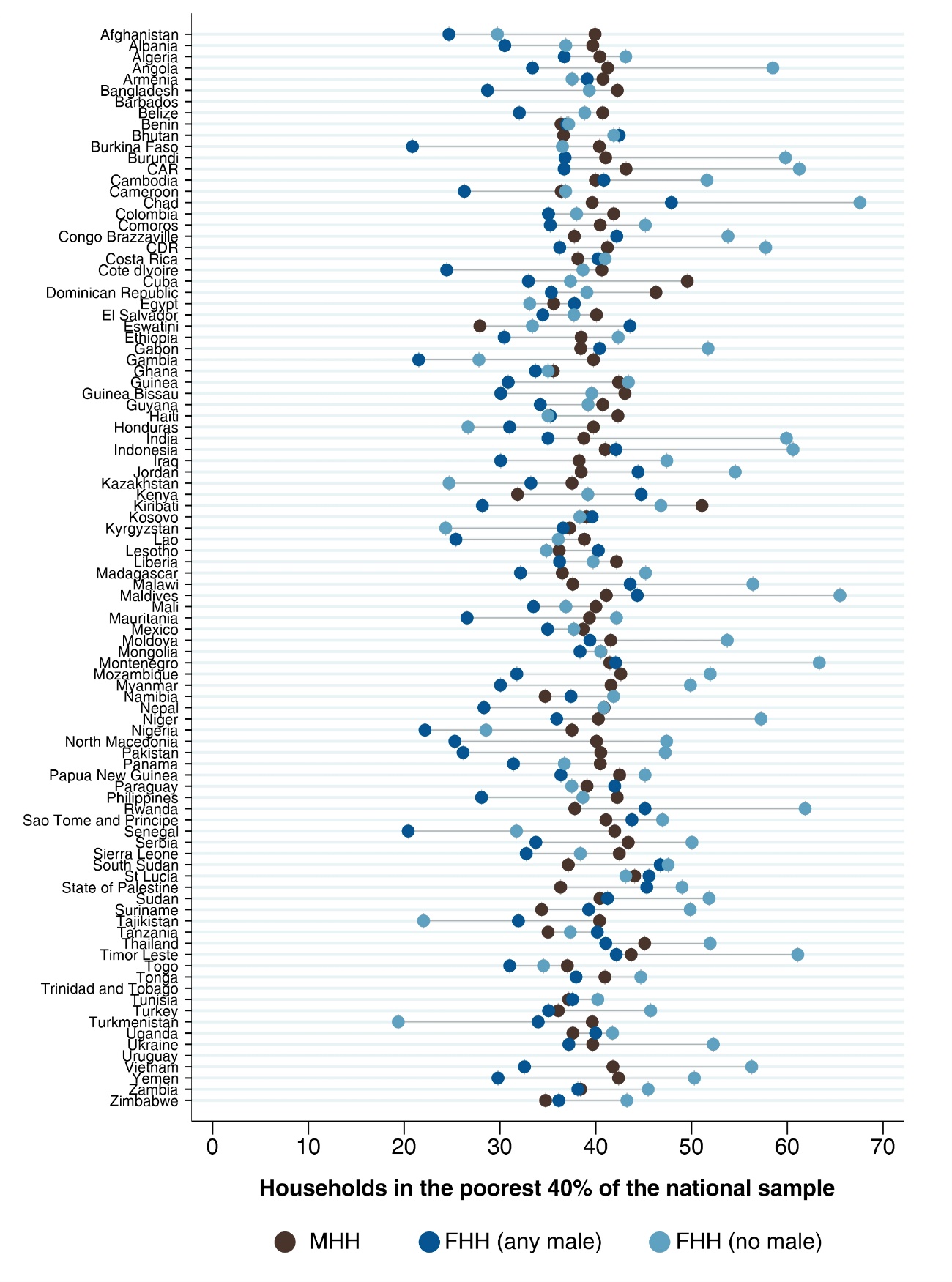


*Note: For each of the three categories of sex of the head of household in a given country, we show the proportions of households in the two poorest quintiles, or the poorest 40% of all households.*


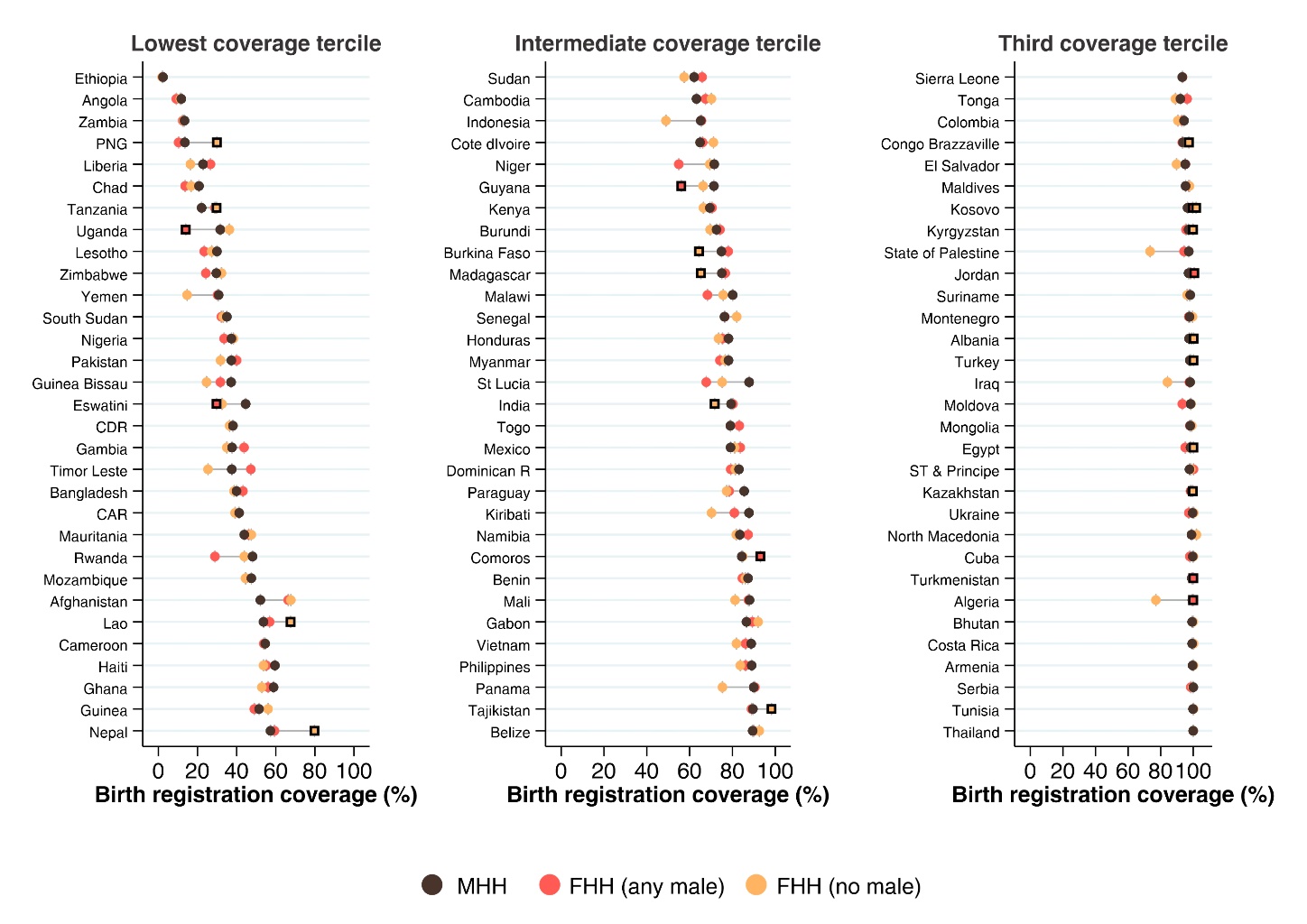


Square symbols identify FHH groups that are significantly (P<0.05) different from the MHH group. Circles identify FHH groups for which the differences from MHH were not significant.

Countries with N<25 in FHH (any male): Kosovo, Montenegro, St Lucia, State of Palestine, Tunisia

Countries with N<25 in FHH (no male): Afghanistan, Algeria, Armenia, Iraq, Jordan, Kiribati, Kosovo, Kyrgyzstan, Montenegro, North Macedonia, Serbia, St Lucia, State of Palestine, Tonga, Turkey, Turkmenistan, Vietnam

N of countries: 93; N of children: 187,234

**Supplementary Figure 2. Unadjusted birth registration coverage according to household types.**

**Supplementary Table 2: Crude coverage of birth registration according to household headship by country.**

|  |  | **MHH** | | | | **FHH (any male)** | | | | | **FHH (no male)** | | | | |
| --- | --- | --- | --- | --- | --- | --- | --- | --- | --- | --- | --- | --- | --- | --- | --- |
| **Country** | **Year** | **Coverage** | **95% Confidence interval** | | **N of children** | **Coverage** | **95% Confidence interval** | | **N of children** | **P-value** | **Coverage** | **95% Confidence interval** | | **N of children** | **P-value** |
|  |  |  | **Lower limit** | **Upper limit** |  |  | **Lower limit** | **Upper limit** |  |  |  | **Lower limit** | **Upper limit** |  |  |
| Afghanistan | 2015 | 52.1% | 48.8% | 55.5% | 5858 | 68.9% | 44.7% | 93.1% | 38 | 0.123 | 63.9% | 38.2% | 89.5% | 17 | 0.327 |
| Albania | 2017 | 97.5% | 95.6% | 99.3% | 433 | 98.8% | 96.3% | 101.2% | 30 | 0.413 | 100.0% | - | - | 28 | 0.009 |
| Algeria | 2018 | 99.4% | 99.1% | 99.8% | 2731 | 100.0% | 100.0% | 100.0% | 91 | 0.001 | 77.4% | 39.5% | 115.3% | 7 | 0.316 |
| Angola | 2015 | 12.3% | 10.2% | 14.3% | 1999 | 10.9% | 6.5% | 15.4% | 381 | 0.576 | 9.0% | 6.5% | 11.5% | 778 | 0.059 |
| Armenia | 2015 | 99.6% | 98.9% | 100.4% | 287 | 100.0% | - | . | 67 | 0.320 | 100.0% | 100.0% | 100.0% | 9 | 0.320 |
| Bangladesh | 2019 | 39.9% | 38.2% | 41.6% | 4244 | 43.4% | 34.6% | 52.2% | 159 | 0.434 | 39.9% | 30.3% | 49.6% | 144 | 0.992 |
| Belize | 2015 | 89.6% | 85.5% | 93.6% | 285 | 90.3% | 80.6% | 100.0% | 60 | 0.890 | 92.4% | 85.1% | 99.6% | 47 | 0.488 |
| Benin | 2017 | 87.0% | 85.1% | 88.9% | 2511 | 85.8% | 79.9% | 91.7% | 155 | 0.682 | 87.7% | 83.8% | 91.6% | 298 | 0.761 |
| Bhutan | 2010 | 99.4% | 98.7% | 100.1% | 861 | 99.7% | 99.3% | 100.1% | 352 | 0.432 | 100.0% | - | - | 35 | 0.091 |
| Burkina_Faso | 2010 | 73.5% | 71.1% | 75.9% | 2799 | 77.2% | 61.5% | 93.0% | 37 | 0.636 | 66.7% | 58.5% | 75.0% | 160 | 0.133 |
| Burundi | 2016 | 73.4% | 70.7% | 76.0% | 2185 | 71.5% | 59.9% | 83.2% | 85 | 0.761 | 69.2% | 63.6% | 74.9% | 404 | 0.175 |
| CAR | 2018 | 41.0% | 37.4% | 44.6% | 1448 | 46.5% | 37.6% | 55.3% | 178 | 0.231 | 35.8% | 28.0% | 43.6% | 174 | 0.267 |
| Cambodia | 2014 | 62.8% | 58.4% | 67.2% | 1201 | 67.4% | 60.4% | 74.5% | 300 | 0.270 | 69.0% | 53.2% | 84.9% | 34 | 0.434 |
| Cameroon | 2018 | 54.9% | 51.0% | 58.8% | 1542 | 64.1% | 53.8% | 74.5% | 161 | 0.086 | 57.2% | 49.4% | 64.9% | 195 | 0.604 |
| Chad | 2019 | 21.9% | 19.8% | 24.1% | 3526 | 20.3% | 12.1% | 28.5% | 175 | 0.707 | 17.6% | 12.5% | 22.7% | 364 | 0.149 |
| Colombia | 2015 | 94.2% | 92.9% | 95.5% | 1747 | 93.3% | 91.0% | 95.7% | 483 | 0.537 | 90.9% | 86.8% | 95.0% | 323 | 0.135 |
| Comoros | 2012 | 84.4% | 80.4% | 88.4% | 464 | 93.5% | 88.9% | 98.1% | 175 | 0.002 | 84.6% | 72.3% | 97.0% | 45 | 0.974 |
| Congo_Brazzaville | 2014 | 93.8% | 92.5% | 95.2% | 1587 | 94.3% | 88.3% | 100.2% | 86 | 0.885 | 95.8% | 93.2% | 98.4% | 218 | 0.156 |
| CDR | 2017 | 39.3% | 34.8% | 43.7% | 3344 | 41.6% | 29.6% | 53.6% | 215 | 0.683 | 30.2% | 23.9% | 36.5% | 795 | 0.012 |
| Costa_Rica | 2011 | 99.4% | 98.3% | 100.6% | 330 | 100.0% | - | - | 81 | 0.313 | 100.0% | 100.0% | 100.0% | 48 | 0.313 |
| Cote_dIvoire | 2016 | 64.2% | 60.5% | 67.9% | 1619 | 74.7% | 60.8% | 88.6% | 63 | 0.120 | 74.2% | 66.6% | 81.9% | 183 | 0.009 |
| Cuba | 2019 | 99.8% | 99.5% | 100.2% | 359 | 97.8% | 93.5% | 102.1% | 310 | 0.360 | 100.0% | - | - | 109 | 0.318 |
| Dominican_Republic | 2014 | 82.6% | 80.3% | 85.0% | 2809 | 81.0% | 75.9% | 86.2% | 623 | 0.572 | 81.5% | 77.1% | 85.9% | 518 | 0.639 |
| Egypt | 2014 | 98.3% | 97.7% | 98.9% | 3164 | 94.8% | 90.1% | 99.5% | 83 | 0.140 | 100.0% | - | - | 25 | 0.000 |
| El_Salvador | 2014 | 95.1% | 93.0% | 97.2% | 901 | 95.2% | 92.1% | 98.3% | 239 | 0.966 | 89.8% | 80.7% | 98.9% | 137 | 0.261 |
| Eswatini | 2014 | 46.9% | 39.3% | 54.4% | 209 | 26.3% | 17.1% | 35.5% | 123 | 0.004 | 32.6% | 22.6% | 42.6% | 152 | 0.043 |
| Ethiopia | 2016 | 2.3% | 1.3% | 3.2% | 1755 | 2.4% | -0.5% | 5.4% | 183 | 0.916 | 2.5% | -1.2% | 6.2% | 246 | 0.910 |
| Gabon | 2012 | 87.3% | 83.0% | 91.6% | 948 | 88.3% | 81.2% | 95.3% | 154 | 0.832 | 90.1% | 83.9% | 96.4% | 288 | 0.456 |
| Gambia | 2018 | 37.6% | 34.3% | 40.9% | 1510 | 44.4% | 31.8% | 57.0% | 102 | 0.265 | 34.7% | 24.0% | 45.4% | 127 | 0.621 |
| Ghana | 2017 | 58.4% | 54.8% | 61.9% | 1276 | 58.2% | 48.0% | 68.5% | 155 | 0.983 | 53.6% | 46.9% | 60.4% | 364 | 0.237 |
| Guinea | 2018 | 56.9% | 53.4% | 60.4% | 1369 | 64.8% | 51.6% | 77.9% | 74 | 0.226 | 60.5% | 49.7% | 71.4% | 98 | 0.499 |
| Guinea_Bissau | 2018 | 36.2% | 33.1% | 39.3% | 1269 | 36.6% | 25.8% | 47.4% | 122 | 0.941 | 29.1% | 16.0% | 42.2% | 82 | 0.344 |
| Guyana | 2014 | 70.6% | 65.2% | 76.0% | 466 | 58.2% | 47.5% | 68.8% | 112 | 0.053 | 66.6% | 50.5% | 82.7% | 58 | 0.634 |
| Haiti | 2016 | 58.6% | 54.4% | 62.8% | 736 | 56.7% | 49.5% | 63.9% | 319 | 0.672 | 53.2% | 45.5% | 60.9% | 217 | 0.220 |
| Honduras | 2011 | 77.9% | 75.5% | 80.2% | 1880 | 76.4% | 71.1% | 81.7% | 293 | 0.616 | 74.1% | 66.5% | 81.7% | 193 | 0.352 |
| India | 2015 | 79.4% | 78.9% | 80.0% | 43571 | 81.9% | 80.3% | 83.5% | 4077 | 0.004 | 66.2% | 63.4% | 69.0% | 1719 | 0.000 |
| Indonesia | 2017 | 64.8% | 62.6% | 67.0% | 3102 | 66.0% | 58.7% | 73.3% | 244 | 0.751 | 43.3% | 28.5% | 58.0% | 81 | 0.020 |
| Iraq | 2018 | 98.1% | 97.4% | 98.7% | 3060 | 97.8% | 95.3% | 100.2% | 195 | 0.794 | 83.3% | 52.1% | 114.6% | 7 | 0.394 |
| Jordan | 2017 | 97.2% | 96.0% | 98.3% | 2070 | 100.0% | 100.0% | 100.0% | 29 | 0.000 | 98.0% | 93.9% | 102.1% | 17 | 0.689 |
| Kazakhstan | 2015 | 98.8% | 98.0% | 99.6% | 816 | 98.3% | 96.5% | 100.2% | 186 | 0.663 | 100.0% | - | - | 35 | 0.004 |
| Kenya | 2014 | 70.4% | 67.9% | 72.9% | 2890 | 66.1% | 58.5% | 73.8% | 322 | 0.316 | 64.7% | 60.4% | 68.9% | 820 | 0.030 |
| Kiribati | 2018 | 87.1% | 83.2% | 91.1% | 340 | 83.0% | 75.2% | 90.7% | 100 | 0.333 | 69.1% | 43.2% | 94.9% | 15 | 0.228 |
| Kosovo | 2019 | 96.5% | 94.3% | 98.7% | 301 | 100.0% | - | - | 17 | 0.003 | 100.0% | - | - | 4 | 0.003 |
| Kyrgyzstan | 2018 | 97.1% | 95.2% | 98.9% | 612 | 95.6% | 89.4% | 101.7% | 110 | 0.651 | 100.0% | 100.0% | 100.0% | 17 | 0.003 |
| Lao | 2017 | 52.6% | 49.5% | 55.7% | 2124 | 68.2% | 60.3% | 76.1% | 179 | 0.000 | 72.4% | 58.6% | 86.1% | 44 | 0.002 |
| Lesotho | 2018 | 29.9% | 22.9% | 36.9% | 339 | 23.2% | 12.5% | 33.9% | 113 | 0.344 | 27.7% | 16.3% | 39.0% | 105 | 0.745 |
| Liberia | 2013 | 19.3% | 15.5% | 23.1% | 1129 | 21.5% | 13.3% | 29.7% | 343 | 0.531 | 16.1% | 9.8% | 22.4% | 191 | 0.411 |
| Madagascar | 2018 | 75.1% | 72.8% | 77.5% | 2268 | 84.8% | 77.4% | 92.2% | 132 | 0.010 | 60.8% | 53.8% | 67.7% | 255 | 0.000 |
| Malawi | 2015 | 75.6% | 73.6% | 77.7% | 2578 | 70.3% | 62.5% | 78.1% | 235 | 0.201 | 73.1% | 68.1% | 78.1% | 521 | 0.358 |
| Maldives | 2016 | 95.1% | 91.0% | 99.2% | 356 | 97.4% | 94.7% | 100.1% | 229 | 0.368 | 99.0% | 96.9% | 101.0% | 73 | 0.103 |
| Mali | 2018 | 87.9% | 85.8% | 89.9% | 1739 | 87.8% | 79.1% | 96.6% | 87 | 0.993 | 80.3% | 73.3% | 87.2% | 163 | 0.036 |
| Mauritania | 2015 | 43.3% | 39.2% | 47.4% | 1353 | 53.6% | 44.2% | 62.9% | 242 | 0.038 | 44.7% | 38.6% | 50.9% | 373 | 0.681 |
| Mexico | 2015 | 79.5% | 74.3% | 84.6% | 1174 | 82.3% | 72.0% | 92.5% | 167 | 0.617 | 80.2% | 67.3% | 93.1% | 100 | 0.915 |
| Moldova | 2012 | 98.4% | 97.0% | 99.9% | 323 | 93.1% | 83.6% | 102.5% | 32 | 0.285 | 98.1% | 94.4% | 101.8% | 35 | 0.875 |
| Mongolia | 2018 | 98.1% | 96.9% | 99.4% | 1066 | 98.2% | 95.9% | 100.5% | 89 | 0.979 | 99.1% | 97.3% | 100.9% | 58 | 0.400 |
| Montenegro | 2013 | 97.7% | 95.3% | 100.1% | 208 | 97.5% | 92.5% | 102.5% | 18 | 0.958 | 100.0% | - | - | 4 | 0.059 |
| Mozambique | 2015 | 47.6% | 42.3% | 53.0% | 694 | 44.9% | 32.4% | 57.4% | 131 | 0.681 | 44.6% | 37.0% | 52.2% | 263 | 0.456 |
| Myanmar | 2015 | 77.6% | 73.8% | 81.5% | 853 | 78.5% | 69.6% | 87.4% | 128 | 0.857 | 71.2% | 53.5% | 88.8% | 46 | 0.492 |
| Namibia | 2013 | 85.8% | 82.1% | 89.6% | 536 | 89.3% | 84.6% | 94.1% | 275 | 0.238 | 85.7% | 80.6% | 90.9% | 300 | 0.972 |
| Nepal | 2019 | 57.3% | 52.9% | 61.7% | 911 | 59.0% | 47.4% | 70.6% | 121 | 0.777 | 80.7% | 73.0% | 88.4% | 121 | 0.000 |
| Niger | 2012 | 67.3% | 63.9% | 70.7% | 2426 | 57.0% | 35.6% | 78.3% | 36 | 0.378 | 62.4% | 54.2% | 70.6% | 239 | 0.275 |
| Nigeria | 2018 | 34.9% | 32.9% | 37.0% | 5759 | 41.4% | 30.3% | 52.5% | 115 | 0.223 | 36.3% | 30.4% | 42.1% | 381 | 0.664 |
| North_Macedonia | 2018 | 98.8% | 97.6% | 100.1% | 232 | 100.0% | - | - | 28 | 0.072 | 100.0% | - | - | 3 | 0.072 |
| Pakistan | 2017 | 34.9% | 30.5% | 39.3% | 2354 | 45.4% | 33.2% | 57.5% | 127 | 0.051 | 32.7% | 18.9% | 46.4% | 95 | 0.767 |
| Panama | 2013 | 89.6% | 86.0% | 93.1% | 829 | 92.0% | 87.1% | 96.9% | 169 | 0.433 | 75.7% | 59.0% | 92.4% | 114 | 0.142 |
| PNG | 2016 | 14.5% | 11.6% | 17.3% | 1518 | 13.3% | 6.4% | 20.2% | 128 | 0.766 | 20.0% | 7.1% | 32.9% | 112 | 0.316 |
| Paraguay | 2016 | 85.5% | 81.2% | 89.7% | 519 | 79.0% | 71.6% | 86.4% | 249 | 0.142 | 74.7% | 59.1% | 90.2% | 57 | 0.212 |
| Philippines | 2017 | 88.6% | 86.1% | 91.1% | 1798 | 86.6% | 77.4% | 95.9% | 227 | 0.683 | 83.5% | 65.9% | 101.1% | 56 | 0.590 |
| Rwanda | 2014 | 48.3% | 44.9% | 51.7% | 1283 | 30.0% | 19.5% | 40.4% | 74 | 0.009 | 41.8% | 35.3% | 48.3% | 250 | 0.086 |
| Sao_Tome_and_Principe | 2019 | 97.8% | 95.3% | 100.3% | 228 | 100.0% | - | - | 63 | 0.090 | 98.0% | 94.0% | 102.0% | 41 | 0.945 |
| Senegal | 2019 | 74.5% | 70.6% | 78.4% | 1016 | 83.6% | 74.4% | 92.7% | 183 | 0.074 | 83.6% | 72.8% | 94.4% | 99 | 0.067 |
| Serbia | 2019 | 100.0% | - | - | 228 | 98.5% | 95.4% | 101.5% | 48 | - | 100.0% | - | - | 4 | - |
| Sierra_Leone | 2019 | 92.5% | 91.0% | 94.1% | 1559 | 93.8% | 90.6% | 97.0% | 242 | 0.459 | 93.5% | 89.9% | 97.0% | 216 | 0.628 |
| South_Sudan | 2010 | 36.7% | 32.8% | 40.5% | 1050 | 31.1% | 25.1% | 37.0% | 345 | 0.125 | 29.0% | 23.6% | 34.4% | 352 | **0.027** |
| St_Lucia | 2012 | 90.1% | 78.5% | 101.8% | 23 | 67.2% | 42.9% | 91.5% | 16 | 0.135 | 73.0% | 42.5% | 103.5% | 14 | 0.347 |
| State_of_Palestine | 2019 | 97.2% | 96.2% | 98.2% | 1235 | 94.8% | 84.7% | 104.8% | 20 | 0.637 | 73.6% | 31.1% | 116.0% | 6 | 0.343 |
| Sudan | 2014 | 62.1% | 58.7% | 65.5% | 2745 | 66.6% | 52.4% | 80.7% | 80 | 0.523 | 57.4% | 47.4% | 67.4% | 141 | 0.380 |
| Suriname | 2018 | 98.6% | 97.3% | 99.9% | 404 | 97.5% | 94.4% | 100.7% | 239 | 0.537 | 95.2% | 90.2% | 100.2% | 111 | 0.207 |
| Tajikistan | 2017 | 89.4% | 87.1% | 91.8% | 916 | 89.3% | 84.6% | 93.9% | 151 | 0.946 | 100.0% | - | - | 26 | 0.000 |
| Tanzania | 2015 | 21.8% | 19.0% | 24.5% | 1731 | 28.9% | 19.7% | 38.1% | 147 | 0.096 | 32.6% | 24.8% | 40.3% | 159 | 0.002 |
| Thailand | 2019 | 100.0% | 99.9% | 100.0% | 1232 | 100.0% | - | - | 630 | 0.318 | 100.0% | - | - | 132 | 0.318 |
| Timor_Leste | 2016 | 37.9% | 34.4% | 41.5% | 1328 | 47.2% | 35.1% | 59.2% | 116 | 0.106 | 24.4% | 11.0% | 37.8% | 59 | 0.115 |
| Togo | 2017 | 79.0% | 75.2% | 82.7% | 863 | 87.7% | 76.0% | 99.4% | 29 | 0.144 | 78.7% | 69.7% | 87.6% | 100 | 0.946 |
| Tonga | 2019 | 92.3% | 87.1% | 97.5% | 195 | 95.8% | 90.0% | 101.5% | 40 | 0.385 | 87.7% | 63.2% | 112.3% | 6 | 0.722 |
| Tunisia | 2018 | 99.9% | 99.6% | 100.1% | 558 | 100.0% | - | - | 23 | 0.318 | 100.0% | - | - | 8 | 0.318 |
| Turkey | 2013 | 97.9% | 96.5% | 99.3% | 649 | 98.5% | 95.6% | 101.5% | 38 | 0.690 | 100.0% | 100.0% | 100.0% | 4 | 0.004 |
| Turkmenistan | 2019 | 99.1% | 98.3% | 100.0% | 520 | 100.0% | - | - | 113 | 0.042 | 100.0% | - | - | 11 | 0.042 |
| Uganda | 2016 | 24.6% | 22.4% | 26.9% | 2393 | 21.2% | 14.9% | 27.5% | 205 | 0.340 | 31.8% | 26.8% | 36.9% | 478 | 0.004 |
| Ukraine | 2012 | 99.5% | 98.5% | 100.5% | 418 | 97.6% | 93.3% | 101.8% | 229 | 0.395 | 100.0% | 100.0% | 100.0% | 61 | 0.314 |
| Vietnam | 2013 | 88.6% | 85.6% | 91.5% | 537 | 86.9% | 80.8% | 93.1% | 141 | 0.628 | 80.5% | 59.8% | 101.1% | 13 | 0.468 |
| Yemen | 2013 | 30.6% | 28.0% | 33.3% | 2909 | 34.8% | 20.8% | 48.9% | 71 | 0.533 | 16.1% | 0.0% | 32.1% | 33 | 0.205 |
| Zambia | 2018 | 13.7% | 11.2% | 16.1% | 1639 | 12.4% | 5.2% | 19.6% | 134 | 0.762 | 11.2% | 7.0% | 15.3% | 259 | 0.347 |
| Zimbabwe | 2019 | 30.9% | 27.2% | 34.6% | 794 | 22.3% | 13.8% | 30.9% | 134 | 0.104 | 29.1% | 22.6% | 35.6% | 261 | 0.637 |

Reference of comparisons: MHH

**Supplementary Table 3: Adjusted coverage of birth registration according to household headship by country.**

|  |  | **MHH** | | | | **FHH (any male)** | | | | | **FHH (no male)** | | | | |
| --- | --- | --- | --- | --- | --- | --- | --- | --- | --- | --- | --- | --- | --- | --- | --- |
| **Country** | **Year** | **Coverage** | **95% Confidence interval** | | **N of children** | **Coverage** | **95% Confidence interval** | | **N of children** | **P-value** | **Coverage** | **95% Confidence interval** | | **N of children** | **P-value** |
|  |  |  | **Lower limit** | **Upper limit** |  |  | **Lower limit** | **Upper limit** |  |  |  | **Lower limit** | **Upper limit** |  |  |
| Afghanistan | 2015 | 52.1% | 49.2% | 55.0% | 5825 | 66.4% | 46.3% | 86.4% | 38 | 0.123 | 67.7% | 38.6% | 96.9% | 16 | 0.237 |
| Albania | 2017 | 97.5% | 95.6% | 99.3% | 432 | 99.0% | 96.4% | 101.5% | 30 | 0.370 | 100.2% | 99.4% | 101.1% | 28 | 0.017 |
| Algeria | 2018 | 99.4% | 99.1% | 99.8% | 2731 | 100.0% | 99.7% | 100.2% | 91 | 0.006 | 77.1% | 39.2% | 114.9% | 7 | 0.309 |
| Angola | 2015 | 11.7% | 8.8% | 14.6% | 1093 | 9.1% | 4.5% | 13.7% | 209 | 0.373 | 11.2% | 6.0% | 16.5% | 380 | 0.876 |
| Armenia | 2015 | 99.6% | 98.9% | 100.4% | 287 | 100.0% | 99.9% | 100.1% | 67 | 0.322 | 100.1% | 99.9% | 100.3% | 9 | 0.326 |
| Bangladesh | 2019 | 39.9% | 38.3% | 41.6% | 4244 | 43.2% | 34.5% | 52.0% | 159 | 0.457 | 38.7% | 29.3% | 48.1% | 144 | 0.797 |
| Belize | 2015 | 89.5% | 85.3% | 93.6% | 282 | 89.9% | 80.3% | 99.6% | 60 | 0.935 | 92.6% | 85.5% | 99.6% | 47 | 0.435 |
| Benin | 2017 | 87.3% | 85.6% | 89.0% | 2494 | 84.7% | 79.0% | 90.3% | 151 | 0.361 | 86.0% | 82.2% | 89.9% | 294 | 0.554 |
| Bhutan | 2010 | 99.3% | 98.6% | 100.1% | 861 | 99.8% | 99.4% | 100.3% | 352 | 0.297 | 100.0% | 99.6% | 100.3% | 35 | 0.102 |
| Burkina_Faso | 2010 | 74.9% | 71.9% | 78.0% | 1393 | 78.0% | 62.2% | 93.9% | 20 | 0.695 | 64.4% | 54.7% | 74.0% | 104 | 0.050 |
| Burundi | 2016 | 72.6% | 69.3% | 75.9% | 1038 | 74.2% | 59.8% | 88.6% | 36 | 0.827 | 69.6% | 62.9% | 76.3% | 244 | 0.429 |
| CAR | 2018 | 41.3% | 38.0% | 44.6% | 1448 | 41.1% | 34.3% | 48.0% | 178 | 0.970 | 39.2% | 31.9% | 46.5% | 174 | 0.624 |
| Cambodia | 2014 | 63.2% | 58.7% | 67.8% | 770 | 67.4% | 58.7% | 76.2% | 201 | 0.390 | 70.2% | 51.9% | 88.5% | 18 | 0.447 |
| Cameroon | 2018 | 54.6% | 50.3% | 58.8% | 814 | 53.9% | 44.2% | 63.6% | 85 | 0.897 | 54.6% | 42.4% | 66.8% | 95 | 0.999 |
| Chad | 2019 | 20.7% | 18.5% | 22.9% | 3173 | 13.7% | 8.0% | 19.3% | 160 | 0.052 | 16.7% | 11.8% | 21.6% | 348 | 0.162 |
| Colombia | 2015 | 94.3% | 93.1% | 95.6% | 1737 | 93.1% | 90.8% | 95.4% | 475 | 0.335 | 90.6% | 86.3% | 94.8% | 310 | 0.105 |
| Comoros | 2012 | 84.4% | 80.3% | 88.4% | 452 | 93.1% | 88.5% | 97.6% | 173 | 0.003 | 84.8% | 72.4% | 97.1% | 45 | 0.951 |
| Congo_Brazzaville | 2014 | 93.7% | 92.4% | 95.0% | 1587 | 93.3% | 87.4% | 99.2% | 86 | 0.900 | 97.3% | 94.9% | 99.8% | 218 | 0.011 |
| CDR | 2017 | 38.1% | 34.5% | 41.7% | 3344 | 38.1% | 28.8% | 47.4% | 215 | 0.998 | 36.4% | 29.0% | 43.8% | 795 | 0.651 |
| Costa_Rica | 2011 | 99.4% | 98.1% | 100.6% | 329 | 100.1% | 99.8% | 100.5% | 81 | 0.320 | 100.2% | 99.7% | 100.7% | 48 | 0.323 |
| Cote_dIvoire | 2016 | 64.9% | 61.7% | 68.1% | 1619 | 66.1% | 54.2% | 78.0% | 63 | 0.845 | 71.1% | 63.9% | 78.4% | 183 | 0.089 |
| Cuba | 2019 | 99.7% | 99.1% | 100.2% | 359 | 97.9% | 93.9% | 101.9% | 310 | 0.352 | 100.1% | 99.7% | 100.5% | 109 | 0.259 |
| Dominican_Republic | 2014 | 83.1% | 80.8% | 85.4% | 2806 | 79.2% | 74.1% | 84.4% | 623 | 0.184 | 81.4% | 77.3% | 85.5% | 518 | 0.477 |
| Egypt | 2014 | 98.3% | 97.7% | 98.9% | 3159 | 95.1% | 90.6% | 99.5% | 83 | 0.148 | 100.1% | 99.7% | 100.5% | 24 | 0.000 |
| El_Salvador | 2014 | 95.1% | 93.1% | 97.2% | 901 | 95.2% | 92.1% | 98.3% | 239 | 0.953 | 89.8% | 80.8% | 98.8% | 137 | 0.251 |
| Eswatini | 2014 | 44.6% | 37.3% | 51.9% | 209 | 29.7% | 19.7% | 39.8% | 123 | 0.039 | 32.5% | 22.5% | 42.5% | 151 | 0.082 |
| Ethiopia | 2016 | 2.3% | 1.4% | 3.3% | 1742 | 2.0% | -0.4% | 4.3% | 180 | 0.785 | 2.0% | -1.2% | 5.2% | 245 | 0.852 |
| Gabon | 2012 | 86.6% | 81.3% | 91.9% | 626 | 89.3% | 80.2% | 98.5% | 97 | 0.638 | 92.0% | 86.1% | 97.9% | 202 | 0.181 |
| Gambia | 2018 | 37.6% | 34.3% | 41.0% | 1509 | 43.8% | 31.5% | 56.1% | 102 | 0.302 | 35.0% | 24.1% | 45.8% | 127 | 0.657 |
| Ghana | 2017 | 58.9% | 55.3% | 62.4% | 1276 | 56.1% | 46.3% | 65.8% | 155 | 0.598 | 52.9% | 46.2% | 59.6% | 364 | 0.131 |
| Guinea | 2018 | 51.5% | 47.3% | 55.7% | 680 | 49.1% | 29.7% | 68.4% | 37 | 0.809 | 56.1% | 42.7% | 69.4% | 53 | 0.486 |
| Guinea_Bissau | 2018 | 37.2% | 34.2% | 40.1% | 1269 | 31.7% | 23.0% | 40.4% | 122 | 0.279 | 24.6% | 13.1% | 36.1% | 82 | 0.085 |
| Guyana | 2014 | 71.3% | 66.3% | 76.3% | 466 | 56.1% | 46.1% | 66.2% | 112 | 0.012 | 66.3% | 49.8% | 82.9% | 58 | 0.562 |
| Haiti | 2016 | 59.6% | 55.6% | 63.6% | 722 | 55.2% | 48.5% | 61.8% | 311 | 0.302 | 53.7% | 46.0% | 61.4% | 210 | 0.185 |
| Honduras | 2011 | 78.2% | 75.8% | 80.5% | 1857 | 75.4% | 70.0% | 80.7% | 288 | 0.332 | 73.5% | 66.0% | 81.1% | 188 | 0.250 |
| India | 2015 | 79.4% | 78.9% | 79.9% | 43281 | 80.2% | 78.7% | 81.8% | 4054 | 0.303 | 71.7% | 68.8% | 74.7% | 1710 | 0.000 |
| Indonesia | 2017 | 65.2% | 63.1% | 67.4% | 2909 | 65.6% | 58.4% | 72.7% | 223 | 0.929 | 49.0% | 34.5% | 63.5% | 73 | 0.059 |
| Iraq | 2018 | 98.1% | 97.4% | 98.7% | 3060 | 97.7% | 95.2% | 100.1% | 195 | 0.745 | 84.2% | 52.5% | 115.9% | 7 | 0.426 |
| Jordan | 2017 | 97.2% | 96.0% | 98.3% | 2066 | 100.6% | 99.0% | 102.2% | 29 | 0.001 | 98.3% | 93.9% | 102.7% | 16 | 0.616 |
| Kazakhstan | 2015 | 98.8% | 98.0% | 99.6% | 816 | 98.3% | 96.4% | 100.1% | 186 | 0.628 | 99.7% | 99.2% | 100.3% | 35 | 0.010 |
| Kenya | 2014 | 69.5% | 67.1% | 71.8% | 2869 | 70.5% | 61.8% | 79.2% | 319 | 0.825 | 66.5% | 62.1% | 70.9% | 808 | 0.252 |
| Kiribati | 2018 | 87.8% | 83.9% | 91.7% | 339 | 80.9% | 73.5% | 88.4% | 100 | 0.102 | 70.3% | 45.4% | 95.2% | 15 | 0.225 |
| Kosovo | 2019 | 96.5% | 94.2% | 98.7% | 301 | 99.7% | 98.4% | 101.0% | 17 | 0.019 | 101.8% | 99.4% | 104.2% | 4 | 0.009 |
| Kyrgyzstan | 2018 | 97.1% | 95.2% | 98.9% | 612 | 95.6% | 89.5% | 101.7% | 110 | 0.643 | 99.9% | 99.1% | 100.6% | 17 | 0.001 |
| Lao | 2017 | 53.8% | 51.3% | 56.2% | 2124 | 56.9% | 50.6% | 63.1% | 179 | 0.327 | 67.6% | 53.9% | 81.2% | 44 | 0.032 |
| Lesotho | 2018 | 29.9% | 23.5% | 36.4% | 339 | 23.4% | 13.0% | 33.8% | 113 | 0.327 | 27.2% | 16.4% | 38.0% | 105 | 0.682 |
| Liberia | 2013 | 22.8% | 17.5% | 28.2% | 542 | 26.6% | 17.7% | 35.6% | 173 | 0.423 | 16.4% | 8.4% | 24.3% | 117 | 0.234 |
| Madagascar | 2018 | 75.1% | 73.1% | 77.1% | 2265 | 76.7% | 69.5% | 83.8% | 132 | 0.661 | 65.3% | 58.3% | 72.2% | 255 | 0.012 |
| Malawi | 2015 | 80.1% | 77.0% | 83.3% | 817 | 68.3% | 56.3% | 80.3% | 85 | 0.081 | 75.7% | 68.3% | 83.0% | 204 | 0.279 |
| Maldives | 2016 | 95.3% | 91.6% | 99.0% | 352 | 97.2% | 94.5% | 100.0% | 223 | 0.433 | 97.6% | 94.6% | 100.7% | 72 | 0.164 |
| Mali | 2018 | 88.0% | 86.0% | 90.0% | 1707 | 87.5% | 78.9% | 96.1% | 84 | 0.914 | 81.3% | 74.3% | 88.2% | 156 | 0.061 |
| Mauritania | 2015 | 43.9% | 40.4% | 47.4% | 1349 | 46.2% | 39.0% | 53.4% | 242 | 0.563 | 47.5% | 41.8% | 53.2% | 373 | 0.274 |
| Mexico | 2015 | 79.2% | 73.9% | 84.4% | 1174 | 83.7% | 74.3% | 93.0% | 167 | 0.399 | 81.0% | 68.9% | 93.2% | 100 | 0.780 |
| Moldova | 2012 | 98.4% | 96.8% | 99.9% | 323 | 93.3% | 84.0% | 102.5% | 32 | 0.297 | 98.6% | 96.3% | 101.0% | 35 | 0.865 |
| Mongolia | 2018 | 98.2% | 96.9% | 99.4% | 1066 | 97.9% | 95.6% | 100.3% | 89 | 0.859 | 99.0% | 97.2% | 100.9% | 58 | 0.433 |
| Montenegro | 2013 | 97.7% | 95.3% | 100.1% | 208 | 97.3% | 92.2% | 102.3% | 18 | 0.868 | 99.5% | 98.2% | 100.9% | 4 | 0.100 |
| Mozambique | 2015 | 47.5% | 42.2% | 52.9% | 690 | 44.7% | 31.6% | 57.8% | 127 | 0.680 | 44.5% | 37.2% | 51.9% | 259 | 0.458 |
| Myanmar | 2015 | 78.2% | 74.9% | 81.5% | 828 | 74.2% | 66.4% | 81.9% | 128 | 0.342 | 76.7% | 58.6% | 94.7% | 42 | 0.872 |
| Namibia | 2013 | 83.5% | 77.8% | 89.2% | 242 | 87.3% | 80.5% | 94.2% | 130 | 0.380 | 81.8% | 73.8% | 89.8% | 162 | 0.751 |
| Nepal | 2019 | 57.3% | 53.0% | 61.7% | 911 | 59.3% | 47.6% | 71.1% | 121 | 0.744 | 79.8% | 71.8% | 87.8% | 121 | 0.000 |
| Niger | 2012 | 71.5% | 68.0% | 75.1% | 1173 | 54.9% | 28.5% | 81.4% | 17 | 0.278 | 69.4% | 59.7% | 79.2% | 142 | 0.692 |
| Nigeria | 2018 | 37.3% | 34.8% | 39.9% | 2265 | 33.6% | 17.9% | 49.3% | 58 | 0.667 | 38.3% | 31.4% | 45.2% | 191 | 0.788 |
| North_Macedonia | 2018 | 98.9% | 97.7% | 100.1% | 232 | 99.1% | 98.1% | 100.2% | 28 | 0.268 | 102.0% | 99.6% | 104.3% | 3 | 0.087 |
| Pakistan | 2017 | 37.3% | 31.9% | 42.7% | 884 | 40.0% | 28.2% | 51.8% | 46 | 0.678 | 31.8% | 9.6% | 54.0% | 35 | 0.677 |
| Panama | 2013 | 90.0% | 86.4% | 93.5% | 829 | 90.5% | 85.8% | 95.2% | 169 | 0.866 | 75.4% | 58.4% | 92.3% | 114 | 0.127 |
| PNG | 2016 | 13.5% | 10.2% | 16.8% | 749 | 10.3% | 2.8% | 17.8% | 64 | 0.480 | 29.9% | 7.7% | 52.1% | 58 | 0.042 |
| Paraguay | 2016 | 85.5% | 81.5% | 89.6% | 519 | 78.5% | 71.3% | 85.6% | 249 | 0.099 | 77.3% | 62.8% | 91.8% | 57 | 0.308 |
| Philippines | 2017 | 89.0% | 86.8% | 91.2% | 1678 | 86.1% | 80.8% | 91.5% | 209 | 0.277 | 83.7% | 64.9% | 102.5% | 51 | 0.600 |
| Rwanda | 2014 | 48.1% | 43.9% | 52.4% | 625 | 28.9% | 12.8% | 45.0% | 34 | 0.076 | 43.9% | 34.2% | 53.6% | 114 | 0.446 |
| Sao_Tome_and_Principe | 2019 | 97.7% | 95.1% | 100.3% | 228 | 100.1% | 99.6% | 100.7% | 63 | 0.098 | 98.5% | 94.7% | 102.2% | 41 | 0.750 |
| Senegal | 2019 | 76.3% | 72.4% | 80.2% | 1012 | 76.4% | 69.8% | 83.0% | 183 | 0.978 | 82.0% | 72.8% | 91.3% | 99 | 0.211 |
| Serbia | 2019 | 100.0% | 100.0% | 100.0% | 228 | 98.5% | 95.5% | 101.5% | 48 | 0.329 | 99.8% | 99.2% | 100.3% | 4 | 0.346 |
| Sierra_Leone | 2019 | 93.3% | 91.4% | 95.1% | 876 | 93.5% | 88.8% | 98.1% | 129 | 0.934 | 93.3% | 88.1% | 98.5% | 115 | 0.987 |
| South_Sudan | 2010 | 35.0% | 31.7% | 38.3% | 1050 | 32.2% | 26.7% | 37.7% | 345 | 0.398 | 33.1% | 27.5% | 38.8% | 352 | 0.577 |
| St_Lucia | 2012 | 87.8% | 75.5% | 100.2% | 23 | 67.8% | 43.7% | 91.9% | 16 | 0.161 | 75.2% | 47.1% | 103.3% | 14 | 0.429 |
| State_of_Palestine | 2019 | 97.2% | 96.2% | 98.2% | 1235 | 94.4% | 84.4% | 104.4% | 20 | 0.582 | 73.5% | 31.7% | 115.3% | 6 | 0.334 |
| Sudan | 2014 | 62.1% | 59.1% | 65.1% | 2743 | 65.8% | 53.3% | 78.3% | 80 | 0.547 | 57.5% | 49.2% | 65.8% | 141 | 0.305 |
| Suriname | 2018 | 98.2% | 96.6% | 99.7% | 393 | 97.5% | 94.4% | 100.6% | 235 | 0.679 | 96.3% | 91.5% | 101.0% | 110 | 0.460 |
| Tajikistan | 2017 | 89.6% | 87.3% | 91.8% | 915 | 88.9% | 84.2% | 93.7% | 149 | 0.800 | 98.3% | 96.0% | 100.5% | 26 | 0.000 |
| Tanzania | 2015 | 22.1% | 19.6% | 24.5% | 1726 | 28.9% | 21.3% | 36.4% | 147 | 0.063 | 29.7% | 22.7% | 36.8% | 158 | 0.013 |
| Thailand | 2019 | 100.0% | 99.9% | 100.0% | 1232 | 100.0% | 100.0% | 100.0% | 630 | 0.323 | 100.0% | 100.0% | 100.0% | 132 | 0.334 |
| Timor_Leste | 2016 | 37.5% | 33.9% | 41.2% | 1301 | 47.3% | 35.1% | 59.4% | 115 | 0.092 | 25.4% | 11.3% | 39.5% | 56 | 0.168 |
| Togo | 2017 | 79.1% | 75.3% | 82.8% | 862 | 83.2% | 72.0% | 94.4% | 29 | 0.466 | 79.1% | 70.9% | 87.3% | 100 | 0.992 |
| Tonga | 2019 | 92.1% | 87.1% | 97.1% | 195 | 96.3% | 90.1% | 102.4% | 40 | 0.308 | 89.1% | 63.9% | 114.2% | 6 | 0.814 |
| Tunisia | 2018 | 99.9% | 99.6% | 100.1% | 558 | 100.1% | 99.9% | 100.4% | 23 | 0.328 | 100.2% | 99.8% | 100.6% | 8 | 0.327 |
| Turkey | 2013 | 97.9% | 96.5% | 99.3% | 649 | 98.6% | 95.8% | 101.4% | 38 | 0.643 | 100.1% | 98.7% | 101.5% | 4 | 0.024 |
| Turkmenistan | 2019 | 99.1% | 98.3% | 100.0% | 520 | 100.0% | 99.9% | 100.2% | 113 | 0.049 | 99.7% | 99.0% | 100.5% | 11 | 0.216 |
| Uganda | 2016 | 31.6% | 27.5% | 35.7% | 752 | 14.0% | 3.8% | 24.2% | 46 | 0.029 | 36.3% | 29.1% | 43.5% | 198 | 0.263 |
| Ukraine | 2012 | 99.6% | 98.6% | 100.6% | 418 | 97.3% | 92.7% | 101.9% | 229 | 0.358 | 100.3% | 99.5% | 101.1% | 61 | 0.346 |
| Vietnam | 2013 | 88.8% | 86.0% | 91.6% | 537 | 86.2% | 79.8% | 92.5% | 141 | 0.460 | 81.9% | 61.3% | 102.5% | 13 | 0.533 |
| Yemen | 2013 | 30.8% | 28.2% | 33.4% | 2893 | 30.3% | 18.7% | 41.9% | 70 | 0.927 | 14.7% | -0.4% | 29.8% | 33 | 0.154 |
| Zambia | 2018 | 13.3% | 10.9% | 15.8% | 1626 | 12.4% | 5.5% | 19.3% | 131 | 0.808 | 13.0% | 8.4% | 17.7% | 258 | 0.915 |
| Zimbabwe | 2019 | 29.6% | 26.2% | 33.1% | 794 | 24.3% | 15.8% | 32.8% | 134 | 0.282 | 32.3% | 25.6% | 38.9% | 261 | 0.465 |

Reference of comparisons: MHH

**Supplementary Table 4. Summary of changes in gaps (MHH versus FHH) of birth registration coverage due to adjustment for wealth, education and residence.**

| **Difference in crude analyses** | **Difference in adjusted analyses** | **Countries** |
| --- | --- | --- |
| Both FHH groups< MHH | Only FHH (any male) < MHH | Eswatini |
| Both FHH groups > MHH | Only FHH (no male) > MHH | Lao |
| Both FHH groups > MHH | Only FHH (any male) > MHH | Turkmenistan |
| FHH (any male) > MHH & FHH (no male) < MHH | Only FHH (no male) < MHH | India, Madagascar |
| Only FHH (any male) > MHH | No difference | Mauritania |
| Only FHH (any male) < MHH | No difference | Rwanda |
| Only FHH (any male) < MHH | Only FHH (no male) > MHH | Uganda |
| Only FHH (no male) < MHH | No difference | Congo DR, Indonesia, Kenya, Mali, South Sudan |
| Only FHH (no male) > MHH | No difference | Cote d’Ivoire |
| No difference | Only FHH (no male) < MHH | Guyana |
| No difference | Only FHH (any male) > MHH | Burkina Faso |
| No difference | Only FHH (no male) > MHH | Guyana, Papua New Guinea |

**Supplementary Table 5: Crude and adjusted coverage ratios of birth registration coverage comparing FHH groups with MHH by country.**

|  |  |  | **Crude** | | | | | | **Adjusted** | | | | | |
| --- | --- | --- | --- | --- | --- | --- | --- | --- | --- | --- | --- | --- | --- | --- |
|  |  |  | **FHH (any male)** | | | **FHH (no male)** | | | **FHH (any male)** | | | **FHH (no male)** | | |
| **Country** | **ISO** | **Year** | **Coverage**  **ratio** | **Lower limit** | **Upper limit** | **Coverage**  **ratio** | **Lower limit** | **Upper limit** | **Coverage**  **ratio** | **Lower limit** | **Upper limit** | **Coverage**  **ratio** | **Lower limit** | **Upper limit** |
| Afghanistan | AFG | 2015 | 1.32 | 0.93 | 1.88 | 1.22 | 0.82 | 1.84 | 1.27 | 0.94 | 1.73 | 1.30 | 0.84 | 2.00 |
| Albania | ALB | 2017 | 1.01 | 0.98 | 1.05 | 1.03 | 1.01 | 1.05 | 1.02 | 0.98 | 1.05 | 1.03 | 1.01 | 1.05 |
| Algeria | DZA | 2018 | 1.01 | 1.00 | 1.01 | 0.78 | 0.48 | 1.27 | 1.01 | 1.00 | 1.01 | 0.78 | 0.47 | 1.27 |
| Angola | AGO | 2015 | 0.89 | 0.60 | 1.33 | 0.74 | 0.54 | 1.01 | 0.78 | 0.45 | 1.35 | 0.96 | 0.58 | 1.59 |
| Armenia | ARM | 2015 | 1.00 | 1.00 | 1.01 | 1.00 | 1.00 | 1.01 | 1.00 | 1.00 | 1.01 | 1.00 | 1.00 | 1.01 |
| Bangladesh | BGD | 2019 | 1.09 | 0.88 | 1.34 | 1.00 | 0.78 | 1.28 | 1.08 | 0.88 | 1.33 | 0.97 | 0.76 | 1.24 |
| Belize | BLZ | 2015 | 1.01 | 0.89 | 1.14 | 1.03 | 0.94 | 1.13 | 1.00 | 0.89 | 1.13 | 1.03 | 0.95 | 1.13 |
| Benin | BEN | 2017 | 0.99 | 0.92 | 1.06 | 1.01 | 0.96 | 1.06 | 0.97 | 0.91 | 1.04 | 0.99 | 0.94 | 1.03 |
| Bhutan | BTN | 2010 | 1.00 | 1.00 | 1.01 | 1.01 | 1.00 | 1.01 | 1.01 | 1.00 | 1.01 | 1.01 | 1.00 | 1.01 |
| Burkina_Faso | BFA | 2010 | 1.05 | 0.86 | 1.29 | 0.91 | 0.80 | 1.03 | 1.04 | 0.85 | 1.28 | 0.86 | 0.74 | 1.00 |
| Burundi | BDI | 2016 | 0.98 | 0.83 | 1.15 | 0.94 | 0.87 | 1.03 | 1.02 | 0.84 | 1.25 | 0.96 | 0.86 | 1.06 |
| CAR | CAF | 2018 | 1.13 | 0.92 | 1.39 | 0.87 | 0.69 | 1.11 | 1.00 | 0.84 | 1.19 | 0.95 | 0.77 | 1.17 |
| Cambodia | KHM | 2014 | 1.07 | 0.95 | 1.22 | 1.10 | 0.87 | 1.39 | 1.07 | 0.92 | 1.24 | 1.11 | 0.85 | 1.45 |
| Cameroon | CMR | 2018 | 1.17 | 0.98 | 1.40 | 1.04 | 0.89 | 1.22 | 0.99 | 0.82 | 1.19 | 1.00 | 0.79 | 1.26 |
| Chad | TCD | 2019 | 0.93 | 0.62 | 1.39 | 0.80 | 0.60 | 1.08 | 0.66 | 0.43 | 1.00 | 0.81 | 0.59 | 1.09 |
| Colombia | COL | 2015 | 0.99 | 0.96 | 1.02 | 0.96 | 0.92 | 1.01 | 0.99 | 0.96 | 1.01 | 0.96 | 0.91 | 1.01 |
| Comoros | COM | 2012 | 1.11 | 1.04 | 1.18 | 1.00 | 0.86 | 1.17 | 1.10 | 1.03 | 1.18 | 1.00 | 0.86 | 1.17 |
| Congo_Brazzaville | COG | 2014 | 1.00 | 0.94 | 1.07 | 1.02 | 0.99 | 1.05 | 1.00 | 0.93 | 1.06 | 1.04 | 1.01 | 1.07 |
| Congo_Democratic_Republic | COD | 2017 | 1.06 | 0.80 | 1.40 | 0.77 | 0.63 | 0.94 | 1.00 | 0.78 | 1.28 | 0.96 | 0.78 | 1.16 |
| Costa_Rica | CRI | 2011 | 1.01 | 0.99 | 1.02 | 1.01 | 0.99 | 1.02 | 1.01 | 0.99 | 1.02 | 1.01 | 0.99 | 1.02 |
| Cote_dIvoire | CIV | 2016 | 1.16 | 0.96 | 1.41 | 1.16 | 1.04 | 1.29 | 1.02 | 0.85 | 1.22 | 1.10 | 0.99 | 1.22 |
| Cuba | CUB | 2019 | 0.98 | 0.94 | 1.02 | 1.00 | 1.00 | 1.01 | 0.98 | 0.95 | 1.02 | 1.00 | 1.00 | 1.01 |
| Dominican_Republic | DOM | 2014 | 0.98 | 0.92 | 1.05 | 0.99 | 0.93 | 1.05 | 0.95 | 0.89 | 1.02 | 0.98 | 0.93 | 1.04 |
| Egypt | EGY | 2014 | 0.96 | 0.92 | 1.01 | 1.02 | 1.01 | 1.02 | 0.97 | 0.92 | 1.01 | 1.02 | 1.01 | 1.03 |
| El_Salvador | SLV | 2014 | 1.00 | 0.96 | 1.04 | 0.94 | 0.85 | 1.04 | 1.00 | 0.96 | 1.04 | 0.94 | 0.85 | 1.04 |
| Eswatini | SWZ | 2014 | 0.56 | 0.38 | 0.83 | 0.70 | 0.49 | 0.99 | 0.67 | 0.45 | 0.98 | 0.73 | 0.51 | 1.04 |
| Ethiopia | ETH | 2016 | 1.07 | 0.30 | 3.85 | 1.09 | 0.23 | 5.20 | 0.84 | 0.25 | 2.88 | 0.85 | 0.16 | 4.51 |
| Gabon | GAB | 2012 | 1.01 | 0.91 | 1.12 | 1.03 | 0.95 | 1.12 | 1.03 | 0.91 | 1.17 | 1.06 | 0.97 | 1.16 |
| Gambia | GMB | 2018 | 1.18 | 0.88 | 1.58 | 0.92 | 0.67 | 1.27 | 1.16 | 0.87 | 1.55 | 0.93 | 0.67 | 1.29 |
| Ghana | GHA | 2017 | 1.00 | 0.83 | 1.20 | 0.92 | 0.80 | 1.06 | 0.95 | 0.80 | 1.14 | 0.90 | 0.78 | 1.03 |
| Guinea | GIN | 2018 | 1.14 | 0.92 | 1.40 | 1.06 | 0.89 | 1.27 | 0.95 | 0.65 | 1.41 | 1.09 | 0.86 | 1.39 |
| Guinea_Bissau | GNB | 2018 | 1.01 | 0.75 | 1.37 | 0.80 | 0.51 | 1.27 | 0.85 | 0.64 | 1.14 | 0.66 | 0.41 | 1.06 |
| Guyana | GUY | 2014 | 0.82 | 0.68 | 1.00 | 0.94 | 0.74 | 1.20 | 0.79 | 0.65 | 0.95 | 0.93 | 0.73 | 1.19 |
| Haiti | HTI | 2016 | 0.97 | 0.83 | 1.13 | 0.91 | 0.78 | 1.06 | 0.93 | 0.80 | 1.07 | 0.90 | 0.77 | 1.05 |
| Honduras | HND | 2011 | 0.98 | 0.91 | 1.06 | 0.95 | 0.86 | 1.06 | 0.96 | 0.90 | 1.04 | 0.94 | 0.85 | 1.04 |
| India | IND | 2015 | 1.03 | 1.01 | 1.05 | 0.83 | 0.80 | 0.87 | 1.01 | 0.99 | 1.03 | 0.90 | 0.87 | 0.94 |
| Indonesia | IDN | 2017 | 1.02 | 0.91 | 1.14 | 0.67 | 0.47 | 0.94 | 1.01 | 0.90 | 1.12 | 0.75 | 0.56 | 1.01 |
| Iraq | IRQ | 2018 | 1.00 | 0.97 | 1.02 | 0.85 | 0.58 | 1.24 | 1.00 | 0.97 | 1.02 | 0.86 | 0.59 | 1.25 |
| Jordan | JOR | 2017 | 1.03 | 1.02 | 1.04 | 1.01 | 0.97 | 1.05 | 1.04 | 1.02 | 1.06 | 1.01 | 0.97 | 1.06 |
| Kazakhstan | KAZ | 2015 | 1.00 | 0.98 | 1.02 | 1.01 | 1.00 | 1.02 | 0.99 | 0.97 | 1.02 | 1.01 | 1.00 | 1.02 |
| Kenya | KEN | 2014 | 0.94 | 0.83 | 1.06 | 0.92 | 0.85 | 0.99 | 1.01 | 0.89 | 1.15 | 0.96 | 0.89 | 1.03 |
| Kiribati | KIR | 2018 | 0.95 | 0.86 | 1.05 | 0.79 | 0.54 | 1.16 | 0.92 | 0.84 | 1.02 | 0.80 | 0.56 | 1.15 |
| Kosovo | XKX | 2019 | 1.04 | 1.01 | 1.06 | 1.04 | 1.01 | 1.06 | 1.03 | 1.01 | 1.06 | 1.05 | 1.01 | 1.10 |
| Kyrgyzstan | KGZ | 2018 | 0.98 | 0.92 | 1.05 | 1.03 | 1.01 | 1.05 | 0.98 | 0.92 | 1.05 | 1.03 | 1.01 | 1.05 |
| Lao | LAO | 2017 | 1.30 | 1.14 | 1.47 | 1.38 | 1.13 | 1.68 | 1.06 | 0.95 | 1.18 | 1.26 | 1.02 | 1.55 |
| Lesotho | LSO | 2018 | 0.78 | 0.46 | 1.31 | 0.93 | 0.58 | 1.48 | 0.78 | 0.48 | 1.28 | 0.91 | 0.58 | 1.43 |
| Liberia | LBR | 2013 | 1.12 | 0.79 | 1.58 | 0.84 | 0.55 | 1.28 | 1.17 | 0.80 | 1.70 | 0.72 | 0.41 | 1.24 |
| Madagascar | MDG | 2018 | 1.13 | 1.03 | 1.24 | 0.81 | 0.72 | 0.91 | 1.02 | 0.93 | 1.12 | 0.87 | 0.78 | 0.97 |
| Malawi | MWI | 2015 | 0.93 | 0.83 | 1.04 | 0.97 | 0.90 | 1.04 | 0.85 | 0.71 | 1.02 | 0.94 | 0.85 | 1.05 |
| Maldives | MDV | 2016 | 1.02 | 0.97 | 1.08 | 1.04 | 0.99 | 1.09 | 1.02 | 0.97 | 1.07 | 1.02 | 0.99 | 1.06 |
| Mali | MLI | 2018 | 1.00 | 0.90 | 1.11 | 0.91 | 0.84 | 0.99 | 0.99 | 0.90 | 1.10 | 0.92 | 0.85 | 1.00 |
| Mauritania | MRT | 2015 | 1.24 | 1.01 | 1.51 | 1.03 | 0.88 | 1.21 | 1.05 | 0.89 | 1.25 | 1.08 | 0.94 | 1.24 |
| Mexico | MEX | 2015 | 1.04 | 0.90 | 1.19 | 1.01 | 0.85 | 1.20 | 1.06 | 0.93 | 1.20 | 1.02 | 0.87 | 1.21 |
| Moldova | MDA | 2012 | 0.95 | 0.85 | 1.05 | 1.00 | 0.96 | 1.04 | 0.95 | 0.86 | 1.05 | 1.00 | 0.97 | 1.03 |
| Mongolia | MNG | 2018 | 1.00 | 0.97 | 1.03 | 1.01 | 0.99 | 1.03 | 1.00 | 0.97 | 1.02 | 1.01 | 0.99 | 1.03 |
| Montenegro | MNE | 2013 | 1.00 | 0.94 | 1.06 | 1.02 | 1.00 | 1.05 | 1.00 | 0.94 | 1.05 | 1.02 | 1.00 | 1.04 |
| Mozambique | MOZ | 2015 | 0.94 | 0.71 | 1.25 | 0.94 | 0.79 | 1.11 | 0.94 | 0.70 | 1.26 | 0.94 | 0.79 | 1.11 |
| Myanmar | MMR | 2015 | 1.01 | 0.89 | 1.14 | 0.92 | 0.72 | 1.17 | 0.95 | 0.85 | 1.06 | 0.98 | 0.77 | 1.24 |
| Namibia | NAM | 2013 | 1.04 | 0.97 | 1.11 | 1.00 | 0.93 | 1.07 | 1.05 | 0.95 | 1.16 | 0.98 | 0.87 | 1.11 |
| Nepal | NPL | 2019 | 1.03 | 0.84 | 1.27 | 1.41 | 1.25 | 1.59 | 1.04 | 0.84 | 1.27 | 1.39 | 1.23 | 1.57 |
| Niger | NER | 2012 | 0.85 | 0.58 | 1.23 | 0.93 | 0.81 | 1.06 | 0.77 | 0.48 | 1.24 | 0.97 | 0.84 | 1.13 |
| Nigeria | NGA | 2018 | 1.19 | 0.90 | 1.56 | 1.04 | 0.88 | 1.23 | 0.90 | 0.56 | 1.45 | 1.03 | 0.85 | 1.24 |
| North_Macedonia | MKD | 2018 | 1.01 | 1.00 | 1.02 | 1.01 | 1.00 | 1.02 | 1.00 | 1.00 | 1.01 | 1.03 | 1.00 | 1.07 |
| Pakistan | PAK | 2017 | 1.30 | 1.00 | 1.69 | 0.94 | 0.60 | 1.45 | 1.07 | 0.77 | 1.49 | 0.85 | 0.40 | 1.82 |
| Panama | PAN | 2013 | 1.03 | 0.96 | 1.10 | 0.85 | 0.67 | 1.06 | 1.01 | 0.94 | 1.08 | 0.84 | 0.67 | 1.05 |
| Papua_New_Guinea | PNG | 2016 | 0.92 | 0.53 | 1.60 | 1.38 | 0.73 | 2.62 | 0.76 | 0.36 | 1.62 | 2.21 | 1.03 | 4.75 |
| Paraguay | PRY | 2016 | 0.92 | 0.83 | 1.03 | 0.87 | 0.71 | 1.08 | 0.92 | 0.83 | 1.02 | 0.90 | 0.74 | 1.10 |
| Philippines | PHL | 2017 | 0.98 | 0.88 | 1.09 | 0.94 | 0.76 | 1.17 | 0.97 | 0.91 | 1.03 | 0.94 | 0.75 | 1.18 |
| Rwanda | RWA | 2014 | 0.62 | 0.44 | 0.89 | 0.87 | 0.73 | 1.02 | 0.60 | 0.34 | 1.06 | 0.91 | 0.72 | 1.16 |
| Sao_Tome_and_Principe | STP | 2019 | 1.02 | 1.00 | 1.05 | 1.00 | 0.95 | 1.05 | 1.03 | 1.00 | 1.06 | 1.01 | 0.96 | 1.06 |
| Senegal | SEN | 2019 | 1.12 | 0.99 | 1.27 | 1.12 | 0.99 | 1.27 | 1.00 | 0.90 | 1.12 | 1.07 | 0.96 | 1.20 |
| Serbia | SRB | 2019 | 0.98 | - | - | 1.00 | - | - | 0.99 | 0.96 | 1.02 | 1.00 | 0.99 | 1.00 |
| Sierra_Leone | SLE | 2019 | 1.01 | 0.98 | 1.05 | 1.01 | 0.97 | 1.05 | 1.00 | 0.96 | 1.05 | 1.00 | 0.94 | 1.06 |
| South_Sudan | SSD | 2010 | 0.85 | 0.68 | 1.05 | 0.79 | 0.64 | 0.97 | 0.92 | 0.76 | 1.12 | 0.95 | 0.78 | 1.15 |
| St_Lucia | LCA | 2012 | 0.75 | 0.51 | 1.10 | 0.81 | 0.52 | 1.27 | 0.77 | 0.53 | 1.11 | 0.86 | 0.58 | 1.27 |
| State_of_Palestine | PSE | 2019 | 0.97 | 0.88 | 1.08 | 0.76 | 0.42 | 1.35 | 0.97 | 0.87 | 1.08 | 0.76 | 0.43 | 1.34 |
| Sudan | SDN | 2014 | 1.07 | 0.87 | 1.33 | 0.92 | 0.78 | 1.10 | 1.06 | 0.88 | 1.28 | 0.93 | 0.80 | 1.07 |
| Suriname | SUR | 2018 | 0.99 | 0.96 | 1.02 | 0.97 | 0.91 | 1.02 | 0.99 | 0.96 | 1.03 | 0.98 | 0.93 | 1.03 |
| Tajikistan | TJK | 2017 | 1.00 | 0.94 | 1.06 | 1.12 | 1.09 | 1.15 | 0.99 | 0.94 | 1.05 | 1.10 | 1.06 | 1.13 |
| Tanzania | TZA | 2015 | 1.33 | 0.95 | 1.86 | 1.50 | 1.16 | 1.93 | 1.31 | 0.99 | 1.74 | 1.35 | 1.07 | 1.70 |
| Thailand | THA | 2019 | 1.00 | 1.00 | 1.00 | 1.00 | 1.00 | 1.00 | 1.00 | 1.00 | 1.00 | 1.00 | 1.00 | 1.00 |
| Timor_Leste | TLS | 2016 | 1.24 | 0.95 | 1.62 | 0.64 | 0.37 | 1.11 | 1.26 | 0.96 | 1.65 | 0.68 | 0.39 | 1.18 |
| Togo | TGO | 2017 | 1.11 | 0.96 | 1.28 | 1.00 | 0.89 | 1.12 | 1.05 | 0.92 | 1.21 | 1.00 | 0.90 | 1.12 |
| Tonga | TON | 2019 | 1.04 | 0.95 | 1.13 | 0.95 | 0.72 | 1.26 | 1.04 | 0.96 | 1.14 | 0.97 | 0.73 | 1.28 |
| Tunisia | TUN | 2018 | 1.00 | 1.00 | 1.00 | 1.00 | 1.00 | 1.00 | 1.00 | 1.00 | 1.01 | 1.00 | 1.00 | 1.01 |
| Turkey | TUR | 2013 | 1.01 | 0.97 | 1.04 | 1.02 | 1.01 | 1.04 | 1.01 | 0.98 | 1.04 | 1.02 | 1.00 | 1.04 |
| Turkmenistan | TKM | 2019 | 1.01 | 1.00 | 1.02 | 1.01 | 1.00 | 1.02 | 1.01 | 1.00 | 1.02 | 1.01 | 1.00 | 1.02 |
| Uganda | UGA | 2016 | t0.86 | 0.63 | 1.17 | 1.29 | 1.09 | 1.54 | 0.44 | 0.21 | 0.92 | 1.15 | 0.90 | 1.46 |
| Ukraine | UKR | 2012 | 0.98 | 0.94 | 1.03 | 1.01 | 1.00 | 1.02 | 0.98 | 0.93 | 1.03 | 1.01 | 0.99 | 1.02 |
| Vietnam | VNM | 2013 | 0.98 | 0.91 | 1.06 | 0.91 | 0.70 | 1.18 | 0.97 | 0.90 | 1.05 | 0.92 | 0.72 | 1.19 |
| Yemen | YEM | 2013 | 1.14 | 0.76 | 1.70 | 0.52 | 0.19 | 1.42 | 0.98 | 0.67 | 1.44 | 0.48 | 0.17 | 1.32 |
| Zambia | ZMB | 2018 | 0.91 | 0.49 | 1.68 | 0.82 | 0.54 | 1.24 | 0.93 | 0.52 | 1.67 | 0.98 | 0.64 | 1.48 |
| Zimbabwe | ZWE | 2019 | 0.72 | 0.49 | 1.07 | 0.94 | 0.73 | 1.21 | 0.82 | 0.57 | 1.18 | 1.09 | 0.87 | 1.37 |

Reference of comparisons: MHH
